# The development of novel cytochrome P450 2J2 (CYP2J2) inhibitor and the underlying interaction between inhibitor and CYP2J2

**DOI:** 10.1080/14756366.2021.1896500

**Published:** 2021-03-07

**Authors:** Xiangge Tian, Meirong Zhou, Jing Ning, Xiaopeng Deng, Lei Feng, Huilian Huang, Dahong Yao, Xiaochi Ma

**Affiliations:** a Laboratory of Modern Preparation of TCM, Ministry of Education, Jiangxi University of Traditional Chinese Medicine, Nanchang, China; b School of Pharmaceutical Sciences, Shenzhen Technology University, Shenzhen, China; c Dalian Key Laboratory of Metabolic Target Characterization and Traditional Chinese Medicine Intervention, Dalian Medical University, Dalian, China

**Keywords:** CYP2J2, high-throughput screening approach, docking and molecular dynamics, inhibition kinetic, Piperine, structural modification

## Abstract

Human Cytochrome P450 2J2 (CYP2J2) as an important metabolic enzyme, plays a crucial role in metabolism of polyunsaturated fatty acids (PUFAs). Elevated levels of CYP2J2 have been associated with various types of cancer, and therefore it serves as a potential drug target. Herein, using a high-throughput screening approach based on enzymic activity of CYP2J2, we rapidly and effectively identified a novel natural inhibitor (Piperine, **9a**) with IC_50_ value of 0.44 μM from 108 common herbal medicines. Next, a series of its derivatives were designed and synthesised based on the underlying interactions of Piperine with CYP2J2. As expected, the much stronger inhibitors **9k** and **9l** were developed and their inhibition activities increased about 10 folds than Piperine with the IC_50_ values of 40 and 50 nM, respectively. Additionally, the inhibition kinetics illustrated the competitive inhibition types of **9k** and **9l** towards CYP2J2, and *K*_i_ were calculated to be 0.11 and 0.074 μM, respectively. Furthermore, the detailed interaction mechanism towards CYP2J2 was explicated by docking and molecular dynamics, and our results revealed the residue Thr114 and Thr 315 of CYP2J2 were the critical sites of action, moreover the spatial distance between the carbon atom of ligand methylene and Fe atom of iron porphyrin coenzyme was the vital interaction factor towards human CYP2J2.

## Graphical Abstract

**Figure:**
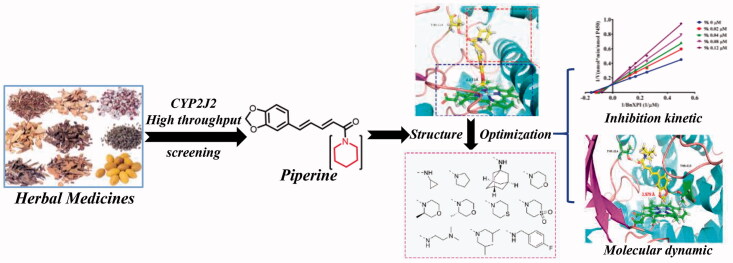

## 1. Introduction

The Cytochromes P450 (CYP450) a major metabolic enzyme family, is mainly located in the endoplasmic reticulum and widely expressed in various organs including liver, intestine, and kidney^1^^,^^2^. It is responsible for the metabolism of various endogenous and exogenous substrates in the presence of cofactor NADPH. Apart from some endogenous substrates, including fatty acids, vitamins, cholesterol, steroids; CYP450 mediates about 90% human drugs metabolism^2^. In human, CYP450 has CYP1, CYP2, and CYP3 families which including different isoforms such as CYP1A1, -1A2, -2A6, -2B6, -2C8, -2C9, -2C19, -2D6, -2E1, -2J2, -3A4/5 and so on. Among these isoforms, CYP2J2 has been paid more and more attention due to its powerful biological function in cardiovascular diseases and cancer development which was owing to its strong metabolism ability for endogenous polyunsaturated fatty acids (PUFAs) such as arachidonic acid (AA) and linoleic acid (LA)^3–8^. For instance, CYP2J2 can metabolise AA to region-isomeric and stereo-selective epoxyeicosatrienoic acids (EETs), including 5,6-EET, 8,9-EET, 11,12-EET and 14,15-EET^4^^,^^9^.

**Figure 1.**
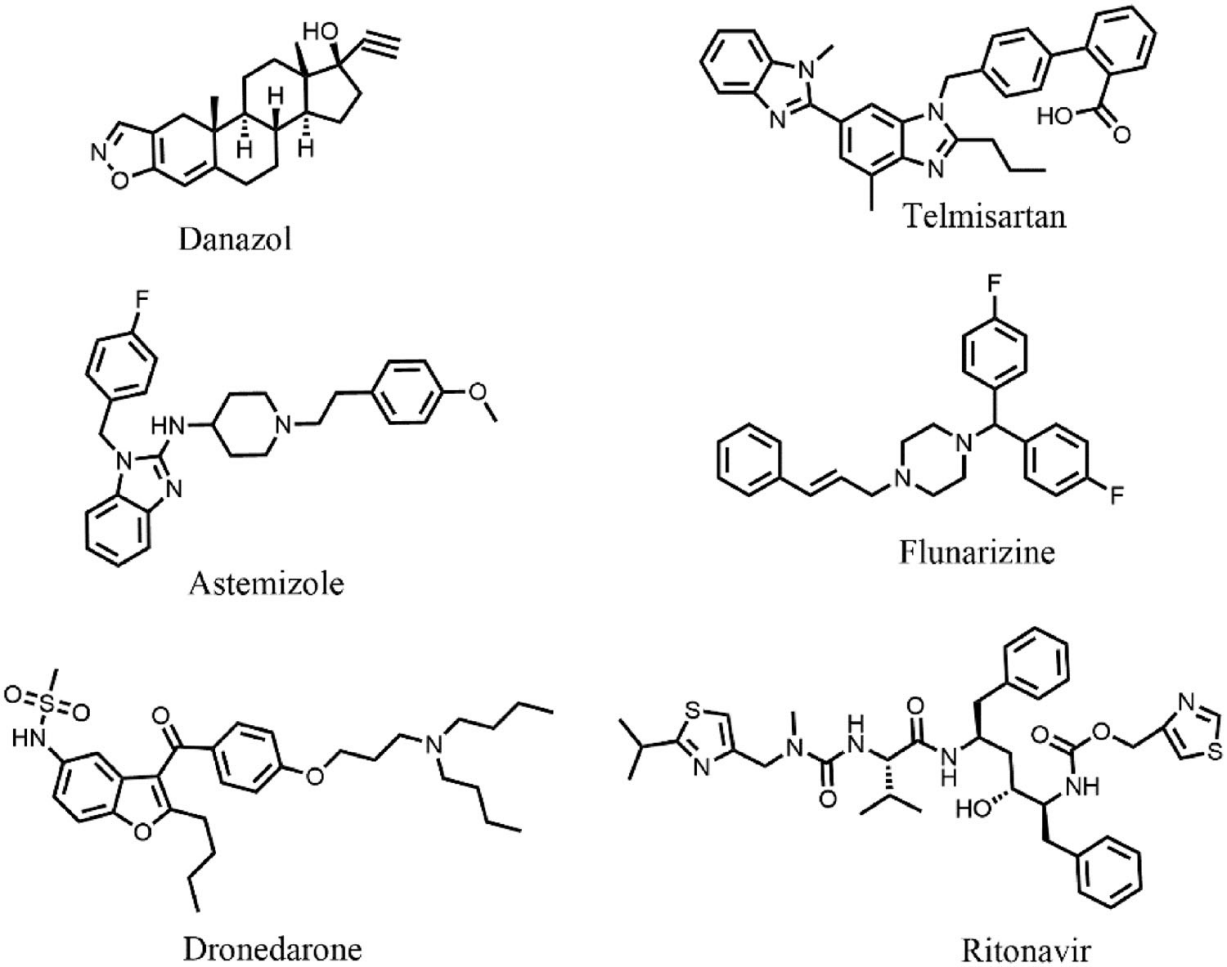
The structures of some reported potent inhibitors for CYP2J2.

Cancer is a leading cause of human death worldwide, many anticancer drugs for the potential targets such epithelial growth factor receptor (EGFR), vascular endothelial growth factor (VEGF), Bcr-Abl, DNA topoisomerase I, Dihydrofolate reductase, Thymoside synthetase and so on were developed; even though the diagnostic and therapeutic techniques have been improved, however, poor prognosis still threatened human life. Recently, the metabolites (EETs) of AA selectively catalysed by CYP2J2 were proved to promote oncogenesis^10–12^. For instance, EETs could significantly promote cell proliferation by the phosphorylation of EGFR and activation of downstream PI3k-AKT and MAPK signalling pathways which had been verified in overexpressing CYP2J2 carcinoma cells. Therefore, CYP2J2 was regarded as a promising antitumor therapeutic target for several malignant tumours^11^^,^^13^. Apart from the therapy of cancer, CYP2J2 also mediated the epoxidation of linoleic acid to form epoxidation of oleic acid (EOA) which can induce the mitochondria dysfunction then increased mortality of burn patients^14^. Several CYP2J2 inhibitors have been described over the past few years^13^, such as Danazol, Telmisartan, Astemizole, Flunarizine, Dronedarone, Ritonavir (Figure 1). Despite the affinity for CYP2J2, most of them displayed a limited selectivity and druggability.

Nowadays, herbal medicines are increasingly employed world wide as alternative and complementary therapies. More importantly, various natural products such as vincristine, camptothecin, artemisinin and paclitaxel, had been isolated and widely used in the clinic^15–18^. For example, CPT-11 as the first-line chemotherapeutic agent, was designed based on the natural product camptothecin^19^^,^^20^. So, herbal medicines were regarded as the valuable resources of drug research and development^16^^,^^21–23^. In our present study, the inhibitory effect of 108 common herbal medicines towards CYP2J2 was screened using our self-developed highly selective fluorescent probe BnXPI of CYP2J2^24^. Among them, *Piper nigrum L*. exhibited prominent inhibition activity and Piperine was identified as the major bioactive component of *Piper nigrum L*. After then, based on the chemical structure of piperine, we conducted the structure optimisation according to the interaction characteristics between Piperine and CYP2J2 catalytic cavity to further design and synthesise a series of Piperine derivatives^24^. Finally, compound **9k** and **9l** were proved to be potent inhibitors of CYP2J2 which both possessed nanomole level of IC_50_ values, and their inhibition kinetics were also further investigated; additionally, the underlying interactions between these potent inhibitors and CYP2J2 enzyme were illustrated by docking and molecular dynamic (MD) simulation. All of our findings would give some useful guidance for development of novel inhibitors of CYP2J2 .

## 2. Materials AND methods

### 2.1. Materials

Human recombinant CYP2J2 was purchased from Cypex (Scotland, UK). *β*-nicotinamide adenine dinucleotide phosphate disodium salt (NADP^+^), D-glucose-6-phosphate, (G-6-P) glucose-6-phosphatedehydrogenase were purchased from Sigma (Darmstadt, Germany). All herbal medicines were obtained from Beijing Tong Ren Tang (China, Beijing). All commercially available reagents and solvents were used as received. ^1^H NMR spectra were recorded at 400 MHz and ^13 ^C NMR data were collected at 100 MHz with complete proton decoupling. ESI-HRMS spectra of all compounds were recorded by Synapt G2-Si^TM^ (Q-TOF-MS) equipped with a high-pressure liquid chromatography (Waters Acquity I-Class^TM^). Flash column chromatography was carried out on silica gel (300–400 mesh, Qingdao Marine Chemical Ltd, Qingdao, China). Thin-layer chromatography (TLC) was performed on TLC silica gel 60 F254 plates. The purities of all final compounds were determined by HPLC to be above 95%.

### 2.2. Method

#### 2.2.1. The inhibitory effect of herbal medicines towards CYP2J2

In our previous study, BnXPI was developed to be the first selective fluorescent probe for CYP2J2^24^. Thus, with the help of the fast and sensitive advantages of fluorescence technology, the potential inhibitory effect of 108 herbal medicines (Table S1) towards CYP2J2 was screened. In brief, in the standard incubation system (100 mM phosphoric acid buffer, pH 7.4, NADPH generate system, BnXPI and extract of herbal medicines), BnXPI was set at 4 μM, and the concentration of CYP2J2 was 0.01 nmol/mL, after the pre-incubation of BnXPI and herbal medicines (final concentration was 20 μg/mL) for 3 min, NADP^+^ was added to initiate the reaction. After additional 30 min (mins) incubation, 100 μL ice acetonitrile was added to terminate the reaction and followed a 20000 g centrifugation at 4 °C, the supernatant was collected and assay on a Synergy H1 Microplate Reader (Bio-Tek). The blank solvent replaces the extract of herbal medicines was set as the control group.

**Table 1.** The CYP2J2 inhibitory activities of compounds 9**a-l.**


Compound	R^1^	Inhibitory activity (IC_50_, μM)^a^
		CYP2J2
9a		0.44 ± 0.05
9b		4.72 ± 0.73
9c		1.58 ± 0.22
9d		9.33 ± 1.06
9e		11.98 ± 1.35
9f		4.66 ± 0.56
9g		2.25 ± 0.56
9h		0.75 ± 0.08
9i		1.24 ± 0.45
9j		11.81 ± 1.47
9k		0.04 ± 0.008
9l		0.05 ± 0.005

^a^Each compound was tested in triplicate; the data are presented as the mean ± SD.

#### 2.2.2. The preparation of fractions of piper nigrum L. and activity assay

In order to discover the active key component that inhibit CYP2J2, the HPLC fractions of *Piper nigrum L*. were further obtained in the preparative high-performance liquid chromatography. The extract of *Piper nigrum L*. was obtained the mobile phase was 10% methanol −90% trifluoroacetic acid water (A) and methanol (B); the flow rate was set at 10 ml/min. The following gradient condition was used: 0 − 15 min 60% A; 15 − 30 min 60 − 44% A; 30 − 40 min 44% A; 40 − 45 min 44–10%A.). Finally, 8 fractions were collected and their inhibitory effects towards CYP2J2 were assayed as mentioned above. The fraction concentration was set at 20 μg/mL, and other conditions were consistent with that mentioned above. After screening, Fr. 5 was selected and its active compound was further isolated and identified by HPLC (Isochromatic conditions methanol: trifluoroacetic acid water = 50:50).

#### 2.2.3. Synthesis

##### 2.2.3.1. Synthesis of intermediate 3

Methyl (E)-4-bromobut-2-enoate (1.79 g, 10.00 mmol) was added to Triethyl phosphite (1.90 ml, 11 mmol), and then the mixture was allowed to warm up for 4 h at 130 °C. Upon the starting material was consumed completely, the hot reaction suspension was poured into 50 ml water, extracted with dichloromethane (3 × 50 ml). The combined organic layers were washed with saturated aqueous sodium bicarbonate and brine, and then dried over anhydrous sodium sulphate. After removing solvent under reduced pressure to obtain the crude oil. The crude oil was purified by silica gel flash chromatography (dichloromethane/methanol 9:1) as a colourless oil, yield 83%. *Methyl (E)-4-(diethoxyphosphoryl)but-2-enoate (****3a****)* ^1^H NMR (400 MHz, CDCl_3_), δ(ppm): 6.89 (1H, m), 5.97 (1H, ddd, *J* = 15.5, 5.0, 1.3 Hz), 4.13 (4H, q, *J* = 6.9 Hz), 3.74 (3H, s), 2.78 (1H, d, *J* = 7.8 Hz), 2.73 (1H, d, *J* = 7.8 Hz).

##### 2.2.3.2. Synthesis of intermediate 6

To a solution of 3, 4-dihydroxybenzaldehyde ***4*** (3.0 g, 10.0 mmol) and K_2_CO_3_ (2.76 g, 20.0 mmol) in DMF (16 ml), and 1, 2-dibromoethane ***5*** (3.76 g, 20.0 mmol) was dropwise added. The reaction mixture was heated at 90 °C and stirred for 12 h. The hot reaction mixture was poured onto ice and extracted with ethyl acetate (3 × 50 ml). The combined organic layers were washed with saturated aqueous sodium bicarbonate and brine, and then dried over anhydrous sodium sulphate. After removing the solvent under reduced pressure, and purified by silica sel flash chromatography (dichloromethane/methanol 2%) as a light yellow solid, yield 73%. Benzo[*d*][1,3]dioxole-5-carbaldehyde (***6***). ^1^H NMR (400 MHz, DMSO-*d*^6^), δ(ppm): 9.81 (1H, s), 7.41 (1H, dd, *J* = 8.0, 1.5 Hz), 7.33 (1H, d, *J* = 1.5 Hz), 6.92 (1H, d, *J* = 8.0 Hz), 6.07 (2H, s); ^13 ^C NMR (100 MHz, CDCl_3_), δ(ppm): 190.2, 153.1, 148.7, 131.9, 128.6, 108.3, 106.9, 102.1;

##### 2.2.3.3. Synthesis of intermediate 7

To a solution of Benzo[d][1,3]dioxole-5-carbaldehyde (***6)*** (0.75 g, 5.0 mmol) and Methyl (E)-4-(diethoxyphosphoryl)but-2-enoate (***3a***) in THF (15 ml), and LiOH (143.7 mg, 6.0 mmol) was added. The reaction mixture was allowed to reflux for 4 h. After removing the solvent under reduced pressure, the mixture was extracted with dichloromethane (3 × 30 ml). The combined organic layers were washed with saturated aqueous sodium bicarbonate and brine, and then dried over anhydrous sodium sulphate. After removing the solvent under reduced pressure, and purified by silica sel flash chromatography (dichloromethane/methanol 2.5%) as a light yellow solid, yield 90%. Methyl (2E,4E)-5-(benzo[*d*][1,3]dioxol-5-yl)penta-2,4-dienoate (***7***) ^1^H NMR (400 MHz, CDCl_3_), δ(ppm): 7.42 (1H, dd, *J* = 15.1, 15.2 Hz), 6.99 (1H, d, *J* = 1.3 Hz), 6.91 (1H, dd, *J* = 8.1, 1.3 Hz), 6.80 (1H, d, *J* = 15.4 Hz), 6.78 (1H, d, *J* = 8.1 Hz), 6.70 (1H, dd, *J* = 15.4, 15.2 Hz), 5.98 (2H,s), 5.94 (1H, d, *J* = 15.1 Hz), 3.76 (3H, s); ^13 ^C NMR (100 MHz, CDCl_3_), δ(ppm): 167.6, 148.6, 148.3, 144.9, 140.2, 130.5, 130.5, 124.5, 122.9, 119.9, 108.5, 105.8, 101.3, 51.4;

##### 2.2.3.4. Synthesis of intermediate 8

Methyl (2E,4E)-5-(benzo[*d*][1,3]dioxol-5-yl)penta-2,4-dienoate ***7*** (0.70 mg, 3.0 mmol) was solved in 1 N NaOH (50% methanol, 10 ml), and the reaction mixture was stirred at room temperature for 8 h. After removing the methanol, the aqueous phase was acidified by.1N HCl to pH = 3, and the resulting precipitate was collected by filtration to give the intermediate ***8*** as a yellow solid, yield 94%. *(2E, 4E)-5-(benzo[d][1,3]dioxol-5-yl)penta-2,4-dienoic acid (****9****)* ^1^H NMR (400 MHz, CDCl_3_), δ(ppm): 12.19 (1H, s), 7.30 (1H, ddd, *J* = 15.1, 14.5, 6.7 Hz), 7.23 (1H, d, *J* = 1.3 Hz), 7.00 (1H, dd, *J* = 8.0, 1.3 Hz), 6.98 (1H, d, *J* = 2.84 Hz), 6.96 (1H, s), 6.92 (1H, d, *J* = 8.0 Hz), 6.05 (2H, s), 5.92 (1H, d, *J* = 15.2 Hz), 1.35 (2H, t, *J* = 7.1 Hz); ^13 ^C NMR (100 MHz, CDCl_3_), δ(ppm): 168.1, 148.5, 148.4, 145.1, 140.2, 130.9, 125.3, 123.5, 121.5, 108.9, 106.2, 101.8;

##### 2.2.3.5. Synthesis of intermediate 9a-l

To a solution of intermediate ***8*** (218.2 mg, 1.0 mmol), amines Derivatives (1.0 mmol, 1eq) and DIEA (165.0 μL, 1.5 mmol) in DMF (10 ml), and HBTU (379.2 mg, 1.0 mmol) was added. The mixture was stirred for 8 h at r.t. and the resulting mixture was diluted with water (20 ml) and extracted with dichloromethane (3 × 30 ml). The combined organic layers were washed with saturated aqueous sodium bicarbonate and brine, and then dried over anhydrous sodium sulphate. After removing the solvent under reduced pressure, and purified by silica sel flash chromatography (dichloromethane/methanol 5–10%) as a light yellow solid.

###### 2.2.3.5.1. (2E,4E)-5-(benzo[d][1,3]dioxol-5-yl)-1-(piperidin-1-yl)penta-2,4-dien-1-one (9a)

Light yellow solid, purified with 5% methanol/dichloromethane, yield 93%. ^1^H NMR (400 MHz, CDCl_3_), δ(ppm): 7.40 (1H, ddd, *J* = 15.1, 15.0, 1.6 Hz), 6.97 (1H,d, *J* = 1.5 Hz), 6.89 (1H, dd, *J* = 8.0, 1.5 Hz), 6.77 (1H, d, *J* = 8.0 Hz), 6.76 (1H, d, *J* = 15.0 Hz), 6.75 (1H, d, *J* = 14.6 Hz), 6.43 (1H, d, *J* = 14.6 Hz), 5.97 (2H, s), 3.63 (2H, brs), 3.53 (2H, brs), 1.65 (2H, m), 1.59 (4H, m); ^13 ^C NMR (100 MHz, CDCl_3_), δ(ppm): 165.4, 148.2, 148.1, 142.4, 138.1, 131.0, 125.3, 122.4, 120.0, 108.4, 105.6, 101.2, 46.8, 43.2, 26.7, 25.6, 24.6; HRMS (ESI)+ calculated for C_17_H_19_NO_3_, [M + H]^+^: *m/z* 286.1438, found 286.1436; Rf = 0.34 (25%, methanol/dichloromethane).

###### 2.2.3.5.2. (2E,4E)-5-(benzo[d][1,3]dioxol-5-yl)-N-cyclopropylpenta-2,4-dienamide (9b)

Light yellow solid, purified with 5% methanol/dichloromethane, yield 89%. ^1^H NMR (400 MHz, CDCl_3_), δ(ppm): 7.36 (1H, dd, *J* = 15.1, 15.2 Hz), 6.96 (1H, d, *J* = 1.3 Hz), 6.87 (1H, dd, *J* = 8.1, 1.3 Hz), 6.78 (1H, d, *J* = 15.4 Hz), 6.77 (1H, d, *J* = 8.1 Hz), 6.66 (1H, dd, *J* = 15.4, 15.2 Hz), 5.97 (2H, s), 5.86 (1H, d, *J* = 15.1 Hz), 5.81 (1H, brs), 2.82 (1H, m), 0.82 (2H, m), 0.55 (2H, m); ^13 ^C NMR (100 MHz, CDCl_3_), δ(ppm): 167.5, 148.2, 148.2, 141.0, 139.0, 130.8, 124.6, 122.8, 122.6, 108.5, 105.7, 101.3, 22.8, 6.7, 6.7; HRMS (ESI)+ calculated for C_15_H_15_NO_3_, [M + H]^+^: *m/z* 258.1125, found 258.1123; Rf = 0.36 (25%, methanol/dichloromethane).

###### 2.2.3.5.3. (2E,4E)-5-(benzo[d][1,3]dioxol-5-yl)-1-(pyrrolidin-1-yl)penta-2,4-dien-1-one (9c)

Light yellow solid, purified with 5% methanol/dichloromethane, yield 68%.^1^H NMR (400 MHz, CDCl_3_), δ(ppm): 7.43 (1H, dd, *J* = 15.1, 15.2 Hz), 6.98 (1H, d, *J* = 1.3 Hz), 6.89 (1H, dd, *J* = 8.1, 1.3 Hz), 6.78 (1H, d, *J* = 15.4 Hz), 6.77 (1H, d, *J* = 8.1 Hz), 6.71 (1H, dd, *J* = 15.4, 15.2 Hz), 6.25 (1H, d, *J* = 14.6 Hz), 5.97 (2H, s), 3.56 (4H, m), 1.98 (2H, m), 1.88 (2H, m); ^13 ^C NMR (100 MHz, CDCl_3_), δ(ppm): 164.9, 148.2, 148.2, 141.7, 138.7, 131.0, 125.2, 122.5, 121.4, 108.5, 105.7, 101.2, 46.4, 45.9, 26.1, 24.3; HRMS (ESI)+ calculated for C_16_H_17_NO_3_, [M + H]^+^: *m/z* 272.1281, found 272.1282; Rf = 0.31 (25%, methanol/dichloromethane).

###### 2.2.3.5.4. (2E,4E)-N-((3s,5s,7s)-adamantan-1-yl)-5-(benzo[d][1,3]dioxol-5-yl)penta-2,4-dienamide (9d)

Light yellow solid, purified with 5% methanol/dichloromethane, yield 81%. ^1^H NMR (400 MHz, CDCl_3_), δ(ppm): 7.27 (1H, dd, *J* = 15.1, 15.2 Hz), 6.96 (1H, d, *J* = 1.3 Hz), 6.88 (1H, dd, *J* = 8.1, 1.3 Hz), 6.76 (1H, d, *J* = 15.4 Hz), 6.74 (1H, d, *J* = 8.1 Hz), 6.65 (1H, d, *J* = 14.6 Hz), 5.96 (2H, s), 5.85 (1H, d, *J* = 15.0 Hz), 5.24 (1H, s), 2.09 (3H, m), 2.06 (6H, m), 1.69 (6H, m); ^13 ^C NMR (100 MHz, CDCl_3_), δ(ppm): 165.2, 148.2, 148.1, 140.2, 138.3, 130.9, 124.7, 124.7, 122.4, 108.4, 105.7, 101.2, 52.1, 41.7, 41.7, 36.3, 36.3, 29.4, 29.4, 29.4, 29.4, 29.4; HRMS (ESI)+ calculated for C_22_H_25_NO_3_, [M + H]^+^: m/z 352.1907, found 352.1906; Rf = 0.35 (25%, methanol/dichloromethane).

###### 2.2.3.5.5. (2E,4E)-5-(benzo[d][1,3]dioxol-5-yl)-1-morpholinopenta-2,4-dien-1-one (9e)

Light yellow solid, purified with 5% methanol/dichloromethane, yield 78%. ^1^H NMR (400 MHz, CDCl_3_), δ(ppm): 7.45 (1H, dd, *J* = 15.1, 15.2 Hz), 6.98 (1H, d, *J* = 1.3 Hz), 6.89 (1H, dd, *J* = 8.1, 1.3 Hz), 6.78 (1H, d, *J* = 15.4 Hz), 6.77 (1H, d, *J* = 8.1 Hz), 6.71 (1H, dd, *J* = 15.4, 15.2 Hz), 6.36 (1H, d, *J* = 14.6 Hz), 5.97 (2H, s), 3.70 (4H, m), 3.64 (4H, m); ^13 ^C NMR (100 MHz, CDCl_3_), δ(ppm): 165.7, 148.3, 148.2, 143.4, 139.1, 130.8, 125.0, 122.7, 118.7, 108.5, 105.7, 101.3, 66.8, 66.8, 46.1, 42.3; HRMS (ESI)^+^ calculated for C_16_H_17_NO_4_, [M + H]^+^: *m/z* 288.1230, found 288.1227; Rf = 0.28 (25%, methanol/dichloromethane).

###### 2.2.3.5.6. (2E,4E)-5-(benzo[d][1,3]dioxol-5-yl)-1-((R)-3-methylmorpholino)penta-2,4-dien-1-one (9f)

Light yellow solid, purified with 7% methanol/dichloromethane, yield 83%. ^1^H NMR (400 MHz, CDCl_3_), δ(ppm): 7.45 (1H, dd, *J* = 15.1, 15.2 Hz), 6.98 (1H, d, *J* = 1.3 Hz), 6.89 (1H, dd, *J* = 8.1, 1.3 Hz), 6.78 (1H, d, *J* = 15.4 Hz), 6.77 (1H, d, *J* = 8.1 Hz), 6.71 (1H, dd, *J* = 15.4, 15.2 Hz), 6.36 (1H, d, *J* = 14.6 Hz), 5.97 (2H, s), 4.19 (2H, m), 3.93 (2H, d, *J* = 8.1 Hz), 3.73 (1H, d, *J* = 11.1 Hz), 3.63 (1H, dd, *J* = 11.1, 3.1 Hz), 3.47 (1H, td, *J* = 12.2, 3.1 Hz), 1.35 (3H, d, *J* = 7.1 Hz); ^13 ^C NMR (100 MHz, CDCl_3_), δ(ppm): 165.7, 148.3, 148.2, 143.4, 139.0, 130.8, 125.1, 122.6, 118.9, 108.5, 105.7, 101.3, 77.0, 70.9, 67.0, 29.6, 15.3; HRMS (ESI)^+^ calculated for C_17_H_19_NO_4_, [M + H]^+^: *m/z* 302.1382, found 302.1385; Rf = 0.30 (25%, methanol/dichloromethane).

###### 2.2.3.5.7. (2E,4E)-5-(benzo[d][1,3]dioxol-5-yl)-1-((S)-3-methylmorpholino)penta-2,4-dien-1-one (9g)

Light yellow solid, purified with 7% methanol/dichloromethane, yield 73%. ^1^H NMR (400 MHz, CDCl_3_), δ(ppm): 7.45 (1H, dd, *J* = 15.1, 15.2 Hz), 6.98 (1H, d, *J* = 1.3 Hz), 6.89 (1H, dd, *J* = 8.1, 1.3 Hz), 6.78 (1H, d, *J* = 15.4 Hz), 6.77 (1H, d, *J* = 8.1 Hz), 6.71 (1H, dd, *J* = 15.4, 15.2 Hz), 6.36 (1H, d, *J* = 14.6 Hz), 5.97 (2H, s), 4.19 (2H, m), 3.93 (2H, d, *J* = 8.1 Hz), 3.73 (1H, d, *J* = 11.1 Hz), 3.63 (1H, dd, *J* = 11.1, 3.1 Hz), 3.47 (1H, td, *J* = 12.2, 3.1 Hz), 1.35 (3H,d, *J* = 7.1 Hz); ^13 ^C NMR (100 MHz, CDCl_3_), δ(ppm): 165.7, 148.3, 148.2, 143.4, 139.0, 130.8, 125.1, 122.6, 118.9, 108.5, 105.7, 101.3, 77.3, 70.9, 67.0, 29.6, 15.3; HRMS (ESI)+ calculated for C_17_H_19_NO_4_, [M + H]^+^: *m/z* 302.1387, found 302.1385; Rf = 0.30 (25%, methanol/dichloromethane).

###### 2.2.3.5.8. (2E,4E)-5-(benzo[d][1,3]dioxol-5-yl)-1-thiomorpholinopenta-2,4-dien-1-one (9h)

Light yellow solid, purified with 5% methanol/dichloromethane, yield 77%. ^1^H NMR (400 MHz, CDCl_3_), δ(ppm): 7.43 (1H, dd, *J* = 15.1, 15.2 Hz), 6.98 (1H, d, *J* = 1.3 Hz), 6.89 (1H, dd, *J* = 8.1, 1.3 Hz), 6.78 (1H, d, *J* = 15.4 Hz), 6.77 (1H, d, *J* = 8.1 Hz), 6.71 (1H, dd, *J* = 15.4, 15.2 Hz), 6.37 (1H, d, *J* = 14.6 Hz), 5.97 (2H, s), 3.93 (4H, m), 2.65 (4H, m); ^13 ^C NMR (100 MHz, CDCl_3_), δ(ppm): 165.7, 148.3, 148.2, 143.4, 139.0, 130.8, 125.0, 122.6, 119.1, 108.5, 105.7, 101.3, 48.5, 44.9, 28.0, 27.4; HRMS (ESI)+ calculated for C_16_H_17_NO_3_S, [M + H]^+^: *m/z* 304.1002, found 304.1002; Rf = 0.35 (25%, methanol/dichloromethane).

###### 2.2.3.5.9. (2E,4E)-5-(benzo[d][1,3]dioxol-5-yl)-1–(1,1-dioxidothiomorpholino)penta-2,4-dien-1-one (9i)

Light yellow solid, purified with 5% methanol/dichloromethane, yield 91%. ^1^H NMR (400 MHz, CDCl_3_), δ(ppm): 7.44 (1H, dd, *J* = 15.1, 15.2 Hz), 6.98 (1H, d, *J* = 1.3 Hz), 6.89 (1H, dd, *J* = 8.1, 1.3 Hz), 6.78 (1H, d, *J* = 15.4 Hz), 6.77 (1H, d, *J* = 8.1 Hz), 6.71 (1H, dd, *J* = 15.4, 15.2 Hz), 6.41 (1H, d, *J* = 14.6 Hz), 5.97 (2H, s), 3.74 (4H, m), 2.00 (4H, m); ^13 ^C NMR (100 MHz, CDCl_3_), δ(ppm): 165.6, 148.3, 148.2, 143.9, 139.3, 130.7, 124.8, 122.7, 118.5, 108.5, 105.7, 101.3, 42.5, 39.0, 34.6, 34.5; HRMS (ESI)+ calculated for C_16_H_17_NO_5_S, [M + H]^+^: *m/z* 336.0900, found 336.0902; Rf = 0.37 (25%, methanol/dichloromethane).

###### 2.2.3.5.10. (2E,4E)-5-(benzo[d][1,3]dioxol-5-yl)-N-(2-(dimethylamino)ethyl)penta-2,4-dienamide (9j)

Light yellow solid, purified with 7% methanol/dichloromethane, yield 69%. ^1^H NMR (400 MHz, CDCl_3_), δ(ppm): 7.35 (1H, dd, *J* = 15.1, 15.2 Hz), 6.97 (1H, d, *J* = 1.3 Hz), 6.88 (1H, dd, *J* = 8.1, 1.3 Hz), 6.78 (1H, d, *J* = 15.4 Hz), 6.77 (1H, d, *J* = 8.1 Hz), 6.71 (1H, dd, *J* = 15.4, 15.2 Hz), 6.42 (1H, t, *J* = 4.6 Hz), 5.97 (2H, s), 5.96 (1H, d, *J* = 14.7 Hz), 3.46 (2H, q, *J* = 5.5 Hz), 2.54 (2H, t, *J* = 6.1 Hz), 2.30 (6H, s); ^13 ^C NMR (100 MHz, CDCl_3_), δ(ppm): 166.2, 148.2, 140.8, 138.7, 130.9, 124.7, 123.2, 122.5, 108.4, 105.7, 101.2, 57.9, 44.9, 36.6, 29.6; HRMS (ESI)+ calculated for C_16_H_20_N_2_O_3_, [M + H]^+^: *m/z* 289.1547, found 289.1545; Rf = 0.37 (25%, methanol/dichloromethane).

###### 2.2.3.5.11. (2E,4E)-5-(benzo[d][1,3]dioxol-5-yl)-N,N-diisobutylpenta-2,4-dienamide (9k)

Light yellow solid, purified with 7% methanol/dichloromethane, yield 83%. ^1^H NMR (400 MHz, CDCl_3_), δ(ppm): 7.43 (1H, dd, *J* = 15.1, 15.2 Hz), 6.99 (1H, d, *J* = 1.3 Hz), 6.89 (1H, dd, *J* = 8.1, 1.3 Hz), 6.78 (1H, d, *J* = 15.4 Hz), 6.77 (1H, d, *J* = 8.1 Hz), 6.71 (1H, dd, *J* = 15.4, 15.2 Hz), 6.39 (1H, t, *J* = 15.1 Hz), 5.97 (2H, s), 3.28 (2H, d, *J* = 7.7 Hz), 3.19 (2H, d, *J* = 7.7 Hz), 2.05 (1H, m), 1.95 (1H, m), 0.93 (6H, d, *J* = 6.5 Hz), 0.89 (6H, d, *J* = 6.5 Hz); ^13 ^C NMR (100 MHz, CDCl_3_), δ(ppm): 166.9, 148.2, 148.1, 142.3, 138.3, 131.0, 125.3, 122.4, 120.5, 108.4, 105.7, 101.2, 77.3, 77.0, 76.7, 56.0, 54.6, 29.6, 28.9, 26.9, 20.2, 20.1; HRMS (ESI)+ calculated for C_20_H_27_NO_3_, [M + H]^+^: *m/z* 330.2064, found 330.2063; Rf = 0.35 (25%, methanol/dichloromethane).

###### 2.2.3.5.12. (2E,4E)-5-(benzo[d][1,3]dioxol-5-yl)-N-(4-fluorobenzyl)penta-2,4-dienamide (9l)

Light yellow solid, purified with 7% methanol/dichloromethane, yield 72%. ^1^H NMR (400 MHz, CDCl_3_), δ(ppm): 7.40 (1H, dd, *J* = 15.1, 15.2 Hz), 7.29 − 7.26 (2H, m), 7.04 − 6.97 (3H, m), 6.89 (1H, dd, *J* = 8.1, 1.3 Hz), 6.78 (1H, d, *J* = 15.4 Hz), 6.77 (1H, d, *J* = 8.1 Hz), 6.71 (1H, dd, *J* = 15.4, 15.2 Hz), 5.97 (2H, s), 5.92 (1H, d, *J* = 15.1 Hz), 5.81 (1H, t, *J* = 6.1 Hz), 4.51 (2H, d, *J* = 5.7 Hz); ^13 ^C NMR (100 MHz, CDCl_3_), δ(ppm): 165.9, 163.4, 160.9, 148.3, 148.2, 141.7, 139.3, 134.1, 134.1, 130.7, 129.5, 129.5, 124.5, 122.7, 122.5, 115.6, 115.4, 108.5, 105.7, 101.3, 43.0; HRMS (ESI)+ calculated for C_19_H_16_FNO_3_, [M + H]^+^: *m/z* 326.1187, found 326.1189. Rf = 0.41 (25%, methanol/dichloromethane).

#### 2.2.4. The inhibition activity of piperine derivatives towards CYP2J2

According to the screening method mentioned above, various concentration of Piperine derivatives were added into our standard incubation system and incubated with CYP2J2, probe BnXPI for 30 min. The concentration of CYP2J2 was set at 3.75 pmol/mL, the concentration of BnXPI was 4 μM. The control group was added blank solvent instead of the inhibitors.

#### 2.2.5. The inhibition kinetic study

In order to clarify the inhibition type of compound **9a**, **9k**, and **9l** towards CYP2J2, the inhibition kinetics were also performed. Briefly, the inhibition activity of target compounds towards the metabolism kinetic of BnPXI (0–8 μM) mediated by CYP2J2 was determinated under the different concentrations of inhibitors. At last, Lineweaver–Burk and Dixon plots were used to fit the data as previously described^25^^,^^26^. The inhibition kinetic type was evaluated by determining the intersection point in the Lineweaver–Burk plots. All the data were fit into the following equation ((Equation (1): Competitive inhibition, Equation (2): Non-competitive inhibition, Equation (3): Uncompetitive inhibition, and Equation (4): Mixed-type inhibition)) to obtain the *K*_i_ values^26^.

(1) $$v=\frac{V_{\max}}{1+\frac{K_{m}}{\left[S\right]}(1+\frac{\left[I\right]}{K_{i}})}$$

(2) $$v=\frac{V_{\max}}{\left(1+\frac{K_{m}}{\left[S\right]})(1+\frac{\left[I\right]}{K_{i}}\right)}$$

(3) $$v=\frac{V_{\max}}{\left(1+\frac{K_{m}}{\left[S\right]}+\frac{\left[I\right]}{K_{i}}\right)}$$

(4) $$v=\frac{V_{\max}}{\frac{K_{m}}{\left[S\right]}(1+\frac{\left[I\right]}{K_{i}})+(1+\frac{\left[I\right]}{\alpha K_{i}})}$$

#### 2.2.6. Molecular docking

To obtain the the model of CYP2J2, we conducted the template search with BLAST against the primary amino acid sequence contained in the SWISS-MODEL template library^27^. For each identified template, the template's quality has been predicted from features of the target-template alignment. The template CYP2R1 (PDB code 3czh) with the highest quality has then been selected for model building. The model were built based on the target-template alignment using discovery studio homology modelling protocol. Coordinates which are conserved between the target and the template are copied from the template to the model. Insertions and deletions are remodelled using a fragment library. Side chains are then rebuilt. Finally, the geometry of the resulting model is regularised by using a Charmm forcefield. The discovery Studio 3.5 was used to perform molecular docking of 9a and 9k within CYP2J2^24^. The binding site was defined as a radius of 8.5 Å. The protein structure was processed by removing water molecules, adding hydrogen atoms and applying Charmm forcefield. The ligands were prepared by adding hydrogen atoms and energy minimisation. Goldscore protocol was used to assess the score of docking, and the other parameters were set as default^28^.

#### 2.2.7. Molecular dynamics (MD) simulations

The MD simulation was performed by Amber 10 package^29^. The first restraining energy minimisation was carried out by the steepest descent method with 0.1 kcal/mol•Å2 restraints for all atoms of the complexes for 5000 steps. And then, we removed the restraints of ligand (only restraining the protein) to perform the second energy minimisation, and another energy minimisation was made under releasing all the restraints. 5000 steps were set for each energy minimisation. To handle the long-range Coulombic interactions, the particle mesh Ewald (PME) summation was used. The SHAKE algorithm was employed on all atoms covalently bonded to a hydrogen atom, allowing for an integration time step of 2 fs in the equilibration and subsequent production runs. The annealed program was from 0 to 310 K for 50 ps. Under releasing all the restraints, the system was again equilibrated for 500 ps. The production phase of the simulations was run without any restraints for a total of 100 ns.

#### 2.2.8. Binding free energy calculation (MM-GBSA)

MM-GBSA calculation was performed using AMBER10 as described^30^. First, we performed the generation of multiple snapshots from an MD trajectory of the protein-ligand complex, stripped of water molecules and counter ions. Snapshots were extracted from the equilibration section of MD trajectory with equally spaced at 10 ps intervals. For each snapshot, the free energy is calculated for each molecular species (complex, protein, and ligand). The binding free energy is computed as the difference:

$$\Delta G_{\text{bind}}=G_{\text{complex}}-G_{\text{protein}}-G_{\text{ligand}}$$

The free energy, G, for each species can be calculated by the following scheme using the MM-GBSA method:

$$G=E_{\text{gas}}+G_{\text{sol}}-TS$$

$$E_{\text{gas}}=E_{\text{int}}+E_{\text{ele}}+E_{\text{vdw}}$$

$$E_{\text{int}}=E_{\text{bond}}+E_{\text{angle}}+E_{\text{torsion}}$$

$$G_{\text{sol}}=G_{\text{GB}}+G_{\text{nonpolar}}$$

$$G_{\text{nopolar}}=\gamma \mathrm{SAS}$$

## 3. Results

### 3.1. Assaying the inhibitory effects of various herbal medicine towards CYP2J2

**Figure 2.**
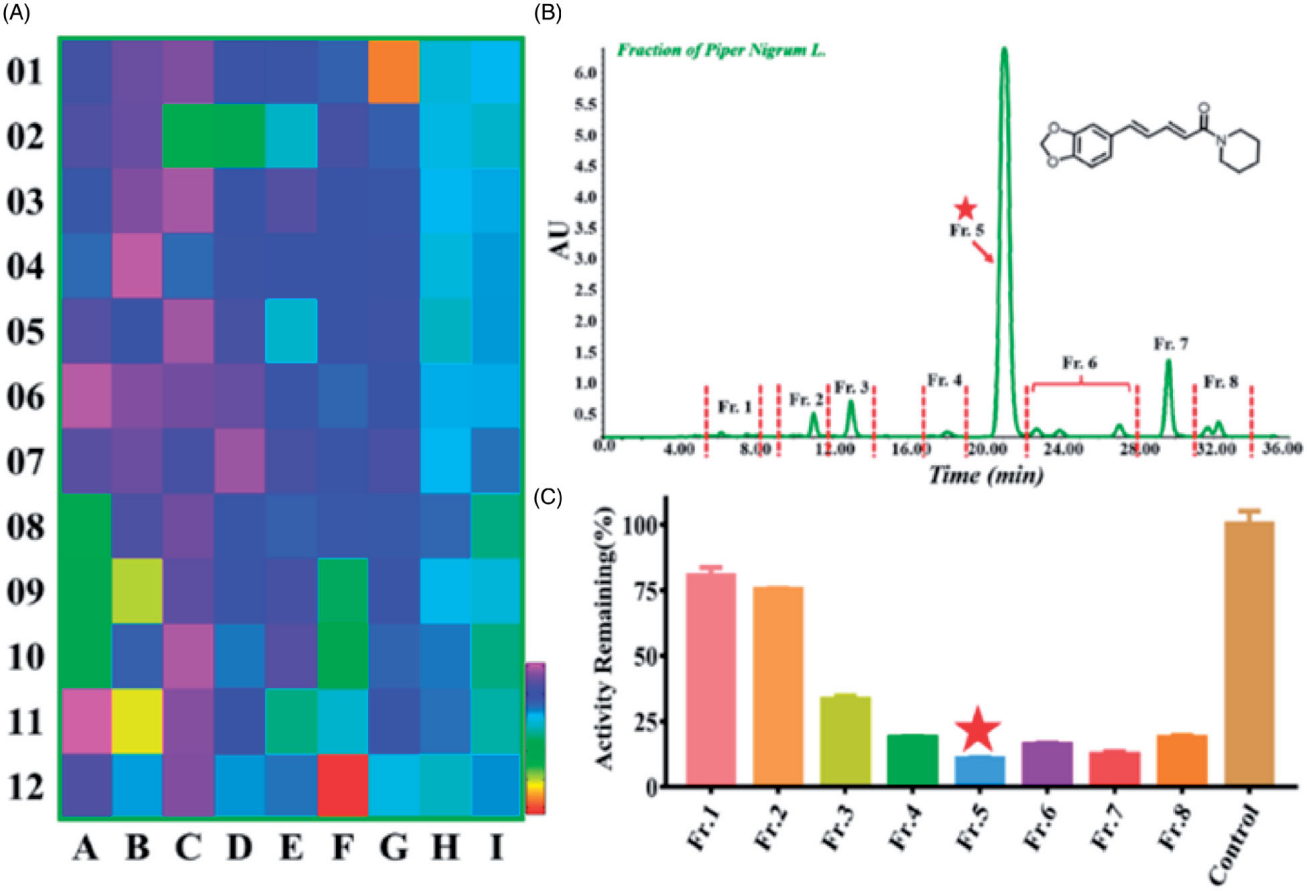
(A) The heat map for the inhibitory effect of 108 herbal medicines towards CYP2J2; (B) The HPLC analysis of the fractions obtained from *Piper nigrum L.*; (C) The inhibition assay of the various fractions of *Piper nigrum L.* against CYP2J2.

In order to discover the novel potent inhibitor for CYP2J2, we screened 108 herbal medicines. As shown in Figure 2(A), the herbal medicines exhibited varying degrees inhibitory effect towards CYP2J2 using the BnXPI as a highly selective probe. Among them, herbal 72 (F12) showed the significant inhibition activity with the residual activity of 15.45%, herbs including 21 (B9), 23 (B11), and 73 (G1) also exhibited good inhibition activity with the residual activity of 40.27%, 37.23%, and 25.69%, respectively; herb 8 (A8), 9 (A9), 10 (A10), 26 (C2), 38 (D2), 69 (F9), 70 (F10) present moderate inhibitory effects with the residual activity from 50%–70%. In contrast, other herbal medicines showed very weak or no inhibition against CYP2J2. Next, herb 72 (F12, *Piper nigrum L.*) was choosed for the further study.

### 3.2. The preparation of HPLC fractions of piper nigrum L. and activity assay

As shown in Figure 2(B), the components of *Piper nigrum L.* were evenly distributed by HPLC analysis, total 8 fractions were obtained according to their polarity. Next, the inhibitory effect of the various fractions towards CYP2J2 was also screened (Figure 2(C)), Fr.4-Fr.8 all exhibited good inhibition activity against CYP2J2; and Fr.3 showed moderate inhibition activity. However, Fr.1 and 2 had very slight inhibitory effects. Among them, Fr. 5 gave more prominent inhibition activity than other fractions. Notably, Fr. 5 was also the major component in *Piper nigrum L.*, and finally a target compound in Fr. 5 was isolated and identified as Piperine using the HPLC and LC-MS/MS analysis by comparing with the standard compound. The IC_50_ value of Piperine towards CYP2J2 was further obtained to be 0.44 μM (Table 1).

### 3.3. Rational design of piperine as CYP2J2 inhibitor

**Figure 3.**
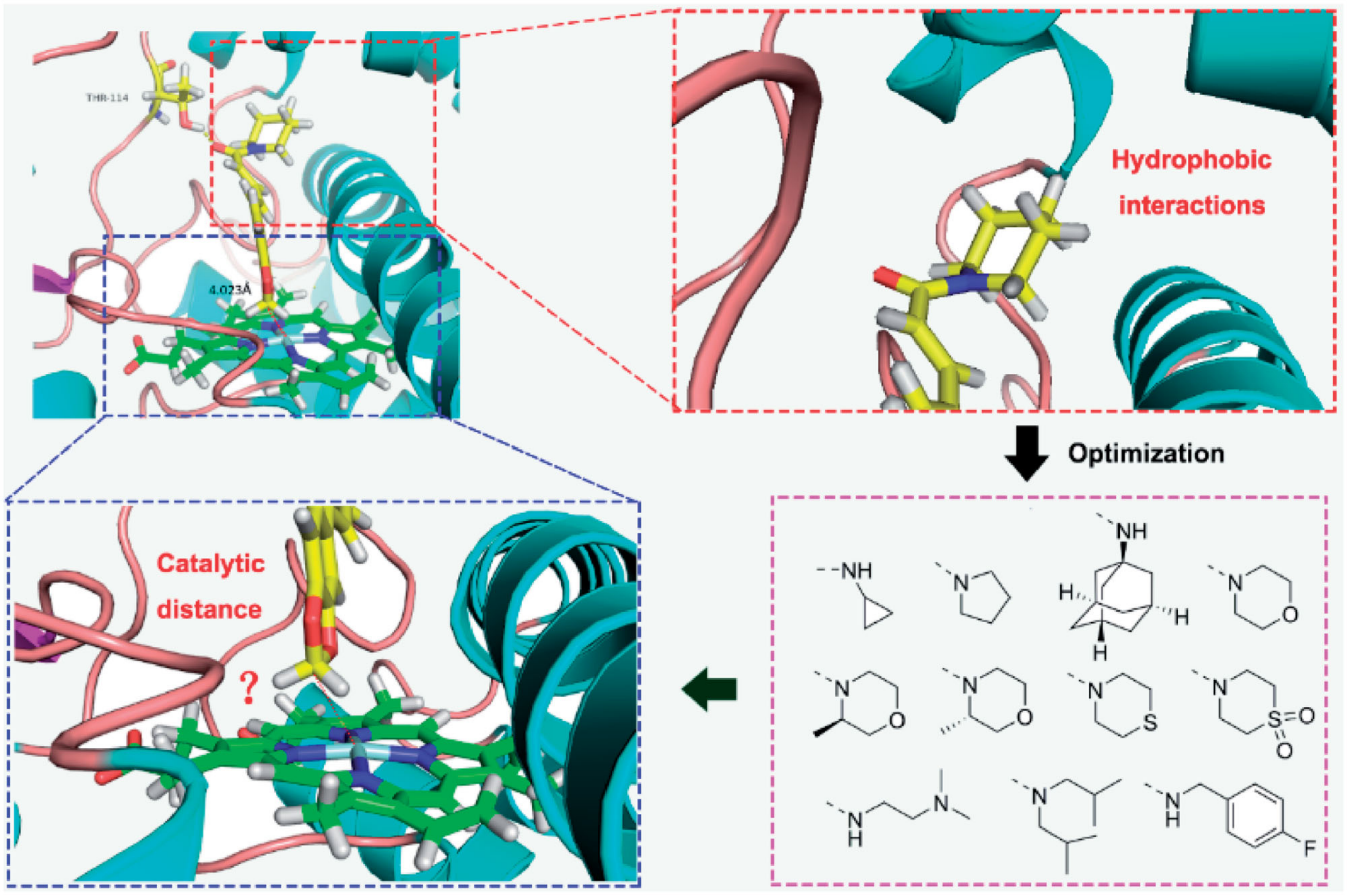
The optimisation of Piperine by structure-based strategy.

To improve the inhibitory potency of Piperine against CYP2J2, we conducted the structure optimisation using structure-based strategy. The docking analysis of Piperine with CYP2J2 indicated that the Piperine occupied the substrate-binding site of CYP2J2, and its methylenedioxy group adopted an active catalytic distance (4.023 Å) to the iron atom of ferriporphyrin which is an indispensable coenzyme for CYP2J2 (Figure 3). Additionally, the terminal piperidine group embedded a big hydrophobic pocket consisting of Phe121, Ile127 and Met128. Given that Piperine is a linear rigid molecule, we could speculate that the potential interactions between terminal substituent groups and the hydrophobic pocket of CYP2J2, play an important role in adoption of active conformations with a suitable catalytic distance. According to the strategy, we designed a series of amine derivatives to displace the piperine group, mainly focussing on the sizes and flexibility of substitution groups.

### 3.4. The synthesis of the piperine derivatives

**Figure 4.**
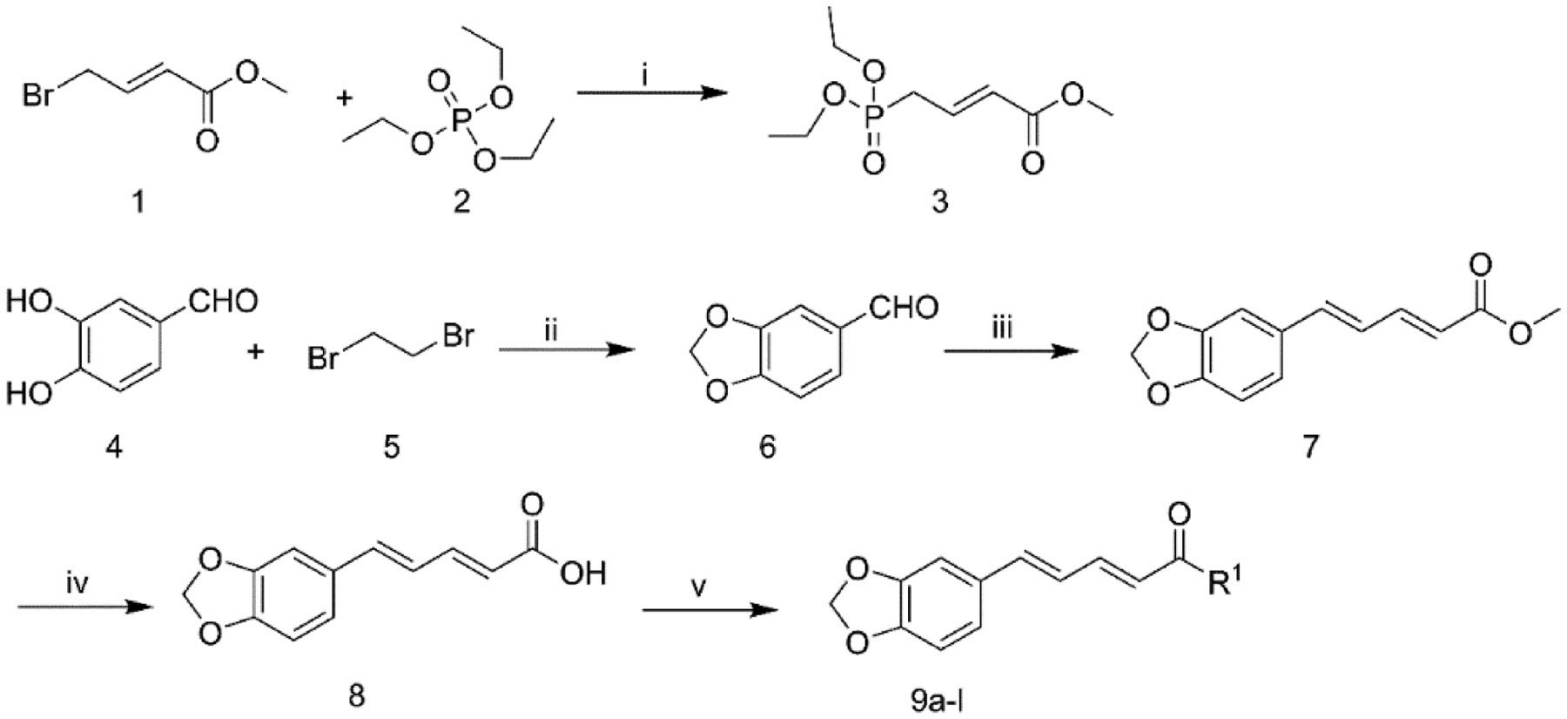
General synthesis of compounds **9a-l.** Reagents and conditions: (i) 130 °C, 4 h; (ii) K_2_CO_3_, DMF, 90 °C, 12 h; (iii) LiOH, THF, reflux, 4 h; (iv) 1 N NaOH (50% methanol); (v) HBTU, DIEA, amines, DMF, r.t.

The synthetic route of compounds **9a-l** was described in Figure 4. The intermediate **3** was prepared by a substitution reaction of methyl (E)-4-bromobut-2-enoate (**1**) with Triethyl phosphate (**2**). The intermediate **6** was prepared by introduction of a methylenedioxy to 3,4-dihydroxybenzaldehyde (**4**). The reaction of intermediate **6** with intermediate **3** yielded intermediate **7** via Wittig reaction, and hydrolysed to yield intermediate **8**. The desired compounds **9a-l** were obtained by reacting intermediate **8** with amines.

### 3.5. The inhibition activity of piperine derivatives towards CYP2J2

**Figure 5.**
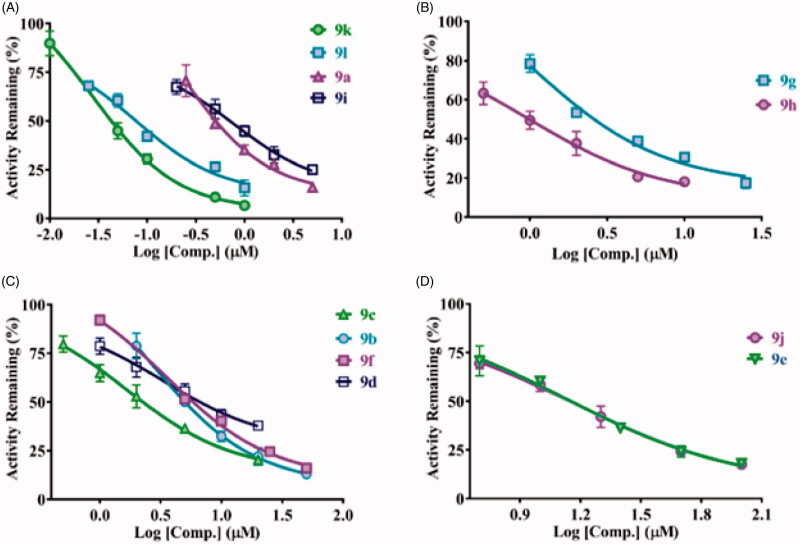
The inhibitory curves of compounds **9a–9l** towards CYP2J2.

Next, we assayed the inhibition activity of piperine derivatives obtained, according to our structure-based synthesis strategy. In brief, as shown in Figure 5, compound **9a-l** exhibited different degrees dose-dependent inhibition behaviour towards CYP2J2. Moreover, the IC_50_ values were obtained, the results demonstrated that the activity has significant distinction (about 300 times gap) ranked from 0.04 to 11.98 μM, and the detailed results was listed in Table 1. Notably, among these compounds**, 9k** and **9l** exhibited nanomolar level inhibition activity, fully indicating that it was reasonable of the structural modification based on the interaction mechanism between Piperine and the key catalytic cavity of CYP2J2.

### 3.6. The inhibition kinetic study of piperine derivatives for CYP2J2

**Figure 6.**
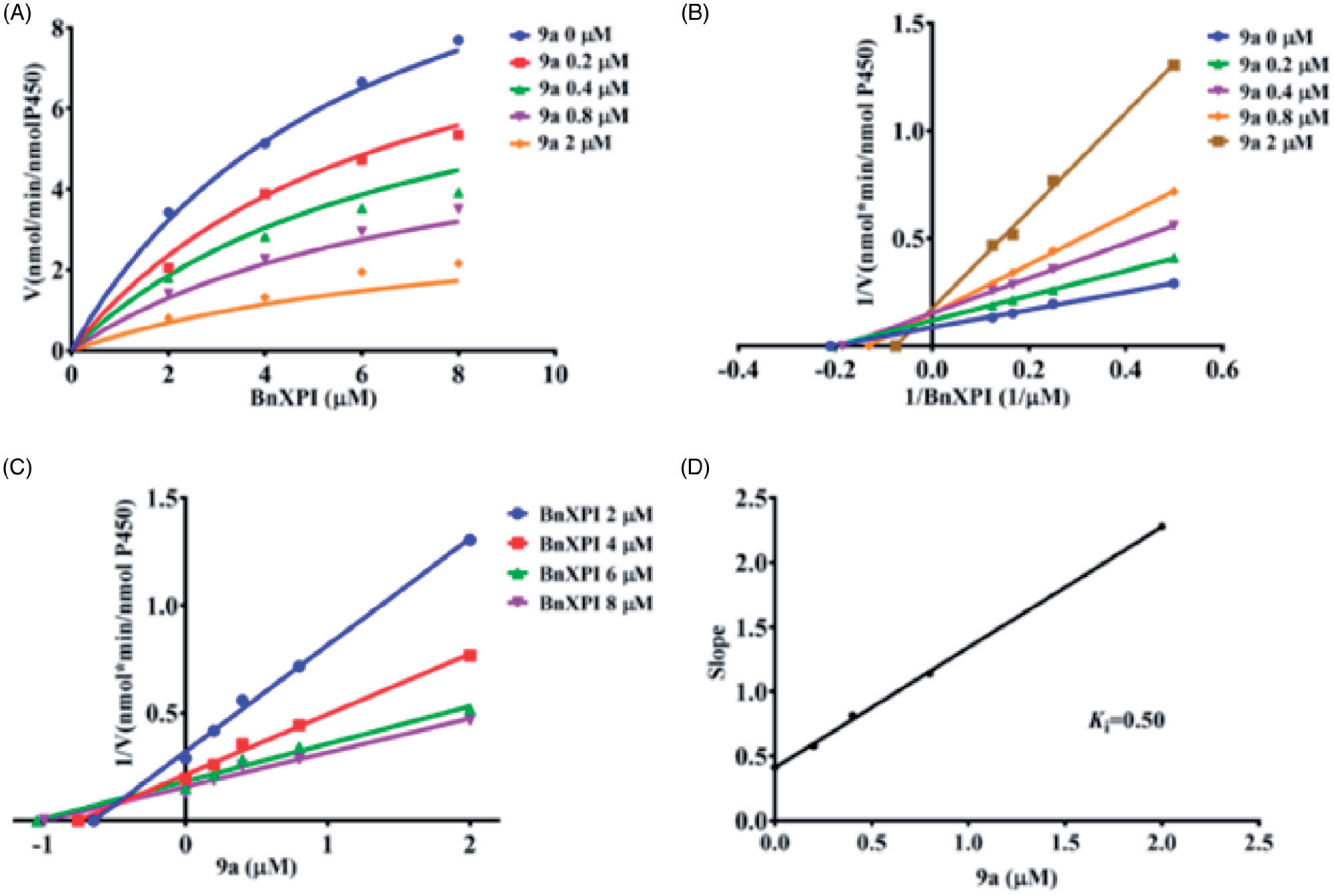
(A) The kinetic curve of BnXPI under the catalysis of CYP2J2 in the presence of different concentration of **9a**; (B) The Lineweaver–Burk plot of the inhibition characteristic of **9a** towards CYP2J2; (C) The Dixon plot of **9a** inhibition behaviour; (D) The Slop curve for the inhibition of CYP2J2 by **9a**. The data points represent the mean value of three experiments.

**Figure 7.**
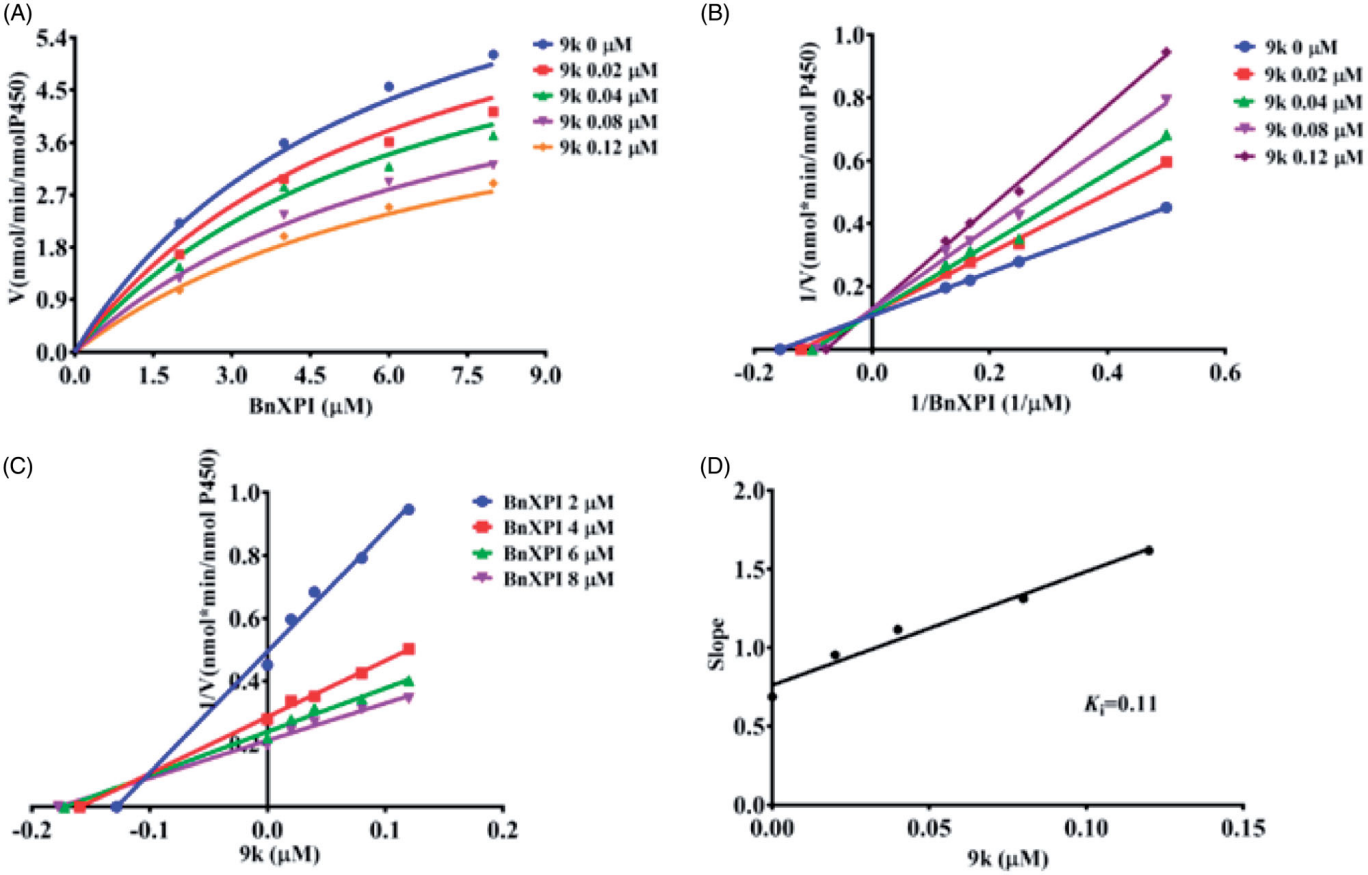
(A) The kinetic curve of BnXPI under the catalysis of CYP2J2 in the presence of different concentration of **9k**; (B) The Lineweaver–Burk plot of the inhibition characteristic of **9k** towards CYP2J2; (C) The Dixon plot of **9k** inhibition behaviour; (D) The Slop curve for the inhibition of CYP2J2 by **9k**. The data points represent the mean value of three experiments.

**Figure 8.**
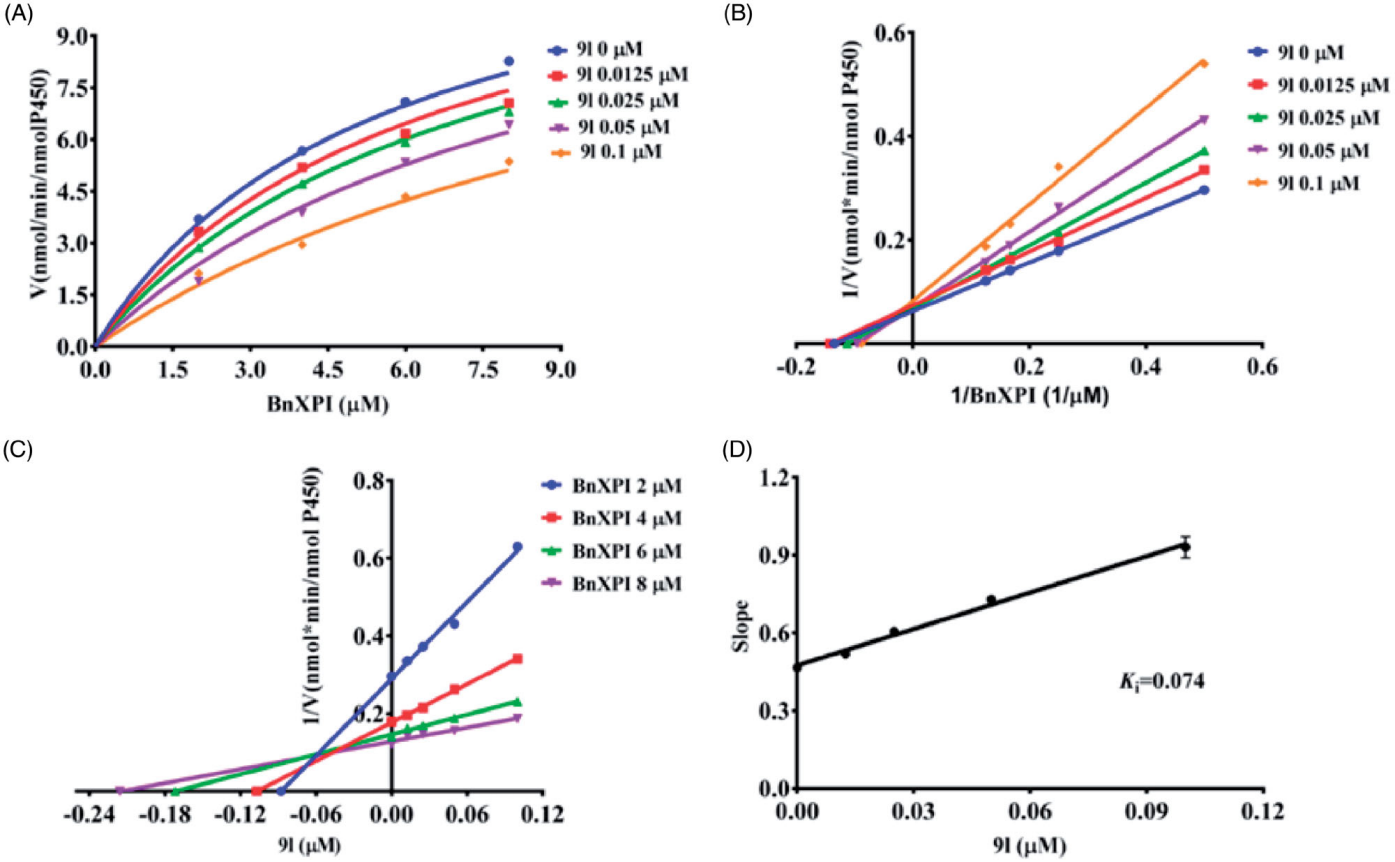
(A) The kinetic curve of BnXPI under the catalysis of CYP2J2 in the presence of different concentration of **9l**; (B) The Lineweaver–Burk plot of the inhibition characteristic of **9l** towards CYP2J2; (C) The Dixon plot of **9l** inhibition behaviour; (D) The Slop curve for the inhibition of CYP2J2 by **9l**. The data points represent the mean value of three experiments.

In order to more comprehensively illustrate the inhibition mechanism of Piperine and its derivatives towards CYP2J2. The inhibition kinetics of **9a**, **9k**, and **9l** were performed, as shown in Figure 6(B), for the kinetic curve, and the intersection in Lineweaver–Burk plot located in the second quadrant, thus the inhibition behaviour was established to be mixed inhibition type. As follows, **9k** and **9l** both exhibited a dose-dependent inhibition on the kinetic curve of BnXPI catalysed by CYP2J2, and the intersection located in Y axis which reflect that both **9k** and **9l** inhibit CYP2J2 were the competitive inhibition model (Figures 7(B) and 8(B)). At last, the inhibition *K*_i_ values were calculated to be 0.50, 0.11, and 0.074 μM for **9a**, **9k** and **9l**, respectively.

### 3.7. Docking and molecular dynamic (MD) simulation

**Figure 9.**
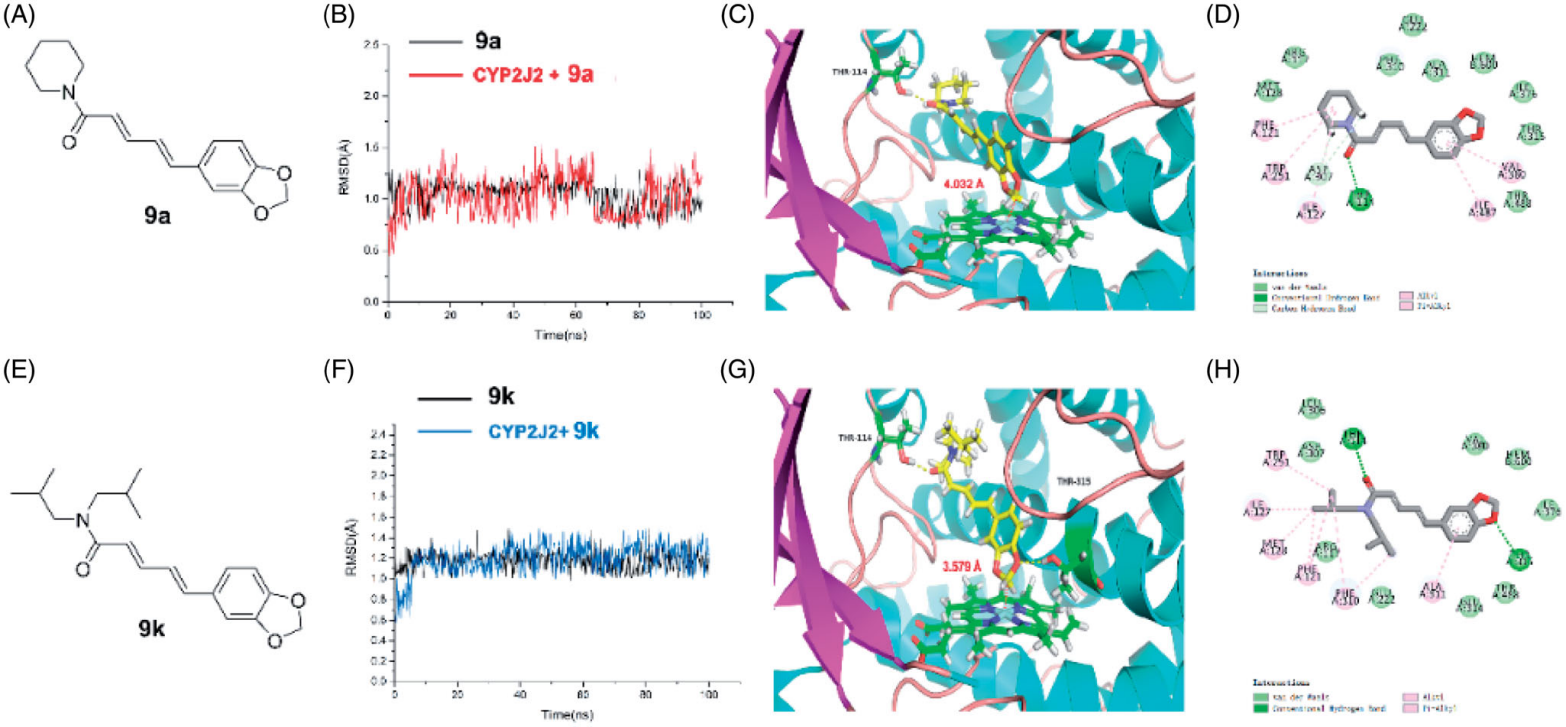
The docking and molecular dynamic (MD) simulation of **9a** and **9k** with CYP2J2. (A, E) The structure of **9a** and **9k**; (B, F) The time evolution of RMSD of backbone atoms for the residues around 5 Å of **9a** and **9k** and heavy atoms of **9a** and **9k**; (C, G) The detailed interactions between **9a** and **9k** with CYP2J2; (D, H) The 2 D interactions of **9a** and **9k** with CYP2J2.

To elucidate the structure basis required for **9k**-bearing potent inhibitory activity against CYP2J2, a computational study including molecular docking, molecular dynamics simulation and binding free energy calculation were performed. Compared to **9a**, the RMSD of **9k**-CYP2J2 fluctuated between 1.0 and 1.4 Å, which indicated that the system was a well-behaved setup. While in the **9a** system, the RMSD presents an extreme volatility between 0.75 and 1.5 Å (Figure 9(B,F)). Additionally, the binding free energy of **9k** system (−48.30 kcal/mol) was lower than that of **9a** system (−36.71 kcal/mol), which suggested that **9k** possessed a higher affinity against CYP2J2 than **9a**. Furthermore, the nonpolar term (−62.47 kcal/mol) played a primary role in **9k** binding to CYP2J2 (Table 2). A detailed view of the interactions was displayed in Figure 9(C,G) 9a and 9k shared a similar binding mode in the catalytic activity centre of CYP2J2. A conserved hydrogen bond initiated by the carbonyl group of **9a** and **9k** and the residue Thr114 was both observed. It is worth noting that **9k** and residue Thr315 formed an additional hydrogen bond, which resulted in a more potent affinity (Figure 9(D,H)). The distance between the carbon atom of ligand methylene and Fe atom of iron porphyrin coenzyme was very important for CYP2J2 inhibition. The catalytic distance of **9k** is closer than that of **9a**. We speculated the key catalytic distance difference caused by the stronger hydrophobic interactions of diisobutylamine group was the structural basis of potent inhibitory activity of **9k**. Collectively, these findings substantiated that **9k** is a novel potent CYP2J2 inhibitor.

**Table 2.** Binding free energy predicted by MM/GBSA methods (kcal/mol).

	9a	9k
ΔE_ele_	−11.12	−14.54
ΔE_vdw_	−49.38	−51.21
ΔE_MM_	−60.50	−65.75
ΔG_sol-np_	−7.39	−11.26
ΔG_sol-ele_	31.18	28.71
ΔG_sol_	23.79	17.45
ΔG_polar_^a^	20.06	14.17
ΔG_nonpolar_^b^	−56.77	−62.47
ΔH_bind_	−36.71	−48.30

^a^ΔG_polar_ = ΔE_ele_ +ΔG_sol-ele_.

^b^ΔG_nonpolar_ = ΔE_vdw_ + ΔG_sol-np_.

## 4. Discussion

As we all know, we have paied much attention to the function of CYP450 for its vital role in the metabolism of lots of clinic drugs^24^^,^^25^^,^^31–33^. However, CYP450 is not only a major metabolic enzyme family in the clinic drugs but also plays a key role in some endogenous substances which are associated with human health^1^^,^^2^^,^^4^^,^^10^^,^^13^^,^^34^. In the previous reports, CYP2J2 is a major isoform responsible for the metabolism of endogenous PUFAs, particularly, previous studies also indicated that it exhibited a significant increase expression in various carcinoma cell lines and could promote proliferation and protect the cells against apoptosis, all of which leads to the inhibition of CYP2J2 became a new therapeutic target for various cancers^9^^,^^13^. However, as far as the substrate, CYP2J2 shares an overlapping substrate spectrum with CYP3A4^35^, Moreover, it lacked highly selective probe for CYP2J2. Thus, the inhibitors for CYP2J2 also exhibited potent inhibition on CYP3A4, such as ketoconazole and danazol^36^^,^^37^. In our previous study, a first selective fluorescent probe (BnXPI) of CYP2J2 was designed and developed, and it exhibited high selectivity towards CYP2J2 among various CYP450 isoforms. Therefore, in the present work, by means of the advantages of fluorescence technology, a high throughput screening method for CYP2J2 inhibitor was established using BnXPI. During the drug development, the bios safe nature is important for its application prospects^38^^,^^39^, herein, after a systemically screening for the herbal medicines, *Piper nigrum L.* as a widely used herbal medicine and daily ingredient was screened out, further Piperine was identified to be a novel potent inhibitor for CYP2J2. In order to improve the inhibitory potency of Piperine against CYP2J2, we conducted a structure optimisation using a structure-based strategy, based on the interaction of Piperine with the substrate-binding site of CYP2J2 and the spatial distance from methylenedioxy group of Piperine Iron atom of ferriporphyrin. As expected, compound **9k** and **9l** both exhibited much stronger inhibition activity towards CYP2J2 compared to Piperine. According to the docking and molecular dynamic (MD) simulation **9k** is closer to the Fe atom of iron porphyrin coenzyme than Piperine and the additional hydrogen bond of **9k** with Thr 315; these interactions made a great contribution to the much better activity of **9k** towards CYP2J2. Notably, in our present work, the *K*_i_ values of compound **9k** and **9l** towards CYP2J2 was not equal to half of the IC_50_ value, the reason was mainly owing to the kinetic behaviour of the probe BnXPI oxidation obey to the sigmoidal kinetic model. During the metabolism, the catalytic progress of BnXPI existing two steps, firstly, the substrate BnXPI (very low concentration) binding with one site of CYP2J2 then induces conformational changes of CYP2J2 that result in altered affinities and the catalytic efficiency, and then near to Michaelis-Menten kinetics. A last, the docking and molecular dynamic (MD) simulation could indicate structural characteristics associated with CYP2J2 inhibition activity, and provided some useful guidance for the development of novel CYP2J2 inhibitors in the further.

## 5. Conclusion

In summary, after the high-throughput screening of the inhibitory effect of 108 herbal medicines towards CYP2J2, *Piper nigrum L.* displayed the most significant inhibition activity, and Piperine as the active constituent in *Piper nigrum L.* was isolated and identified as a novel and natural inhibitor for CYP2J2. According to the underlying interaction of Piperine with CYP2J2 catalytic cavity, a series of Piperine derivatives were designed and synthesised for screening their inhibitory potency against CYP2J2. Finally, compounds **9k** and **9l** both exhibited much stronger inhibition activity towards CYP2J2 than Piperine, and the inhibition type was also revealed to be competitive inhibition by inhibition kinetic analysis. Next, the underlying mechanism of the inhibition was also investigated by docking and molecular dynamic (MD) simulation, the excellent inhibition activity of compound **9k** is owing to its potent affinity with the residue Thr114 and Thr315 in CYP2J2. Additionally, the interaction distance between the carbon atom of ligand methylene and Fe atom of iron porphyrin coenzyme was also proved to be a key factor for effectively developing the potential inhibitor of CYP2J2. Our findings would give some useful guidance for development of CYP2J2 inhibitors in the near future.

## Supplementary Material

Supplemental Material
