# Supplementary material for: The development of novel cytochrome P450 2J2 (CYP2J2) inhibitor and the underlying interaction between inhibitor and CYP2J2
Source: J Enzyme Inhib Med Chem. 2021 Mar 7;36(1):737–48. doi: 10.1080/14756366.2021.1896500 (PMC7946002; doi:10.1080/14756366.2021.1896500)
Supplement: Supplemental Material [file IENZ_A_1896500_SM1163.pdf]

Supporting Information

The development of novel cytochrome P450 2J2 (CYP2J2) inhibitor and the underlying interaction between inhibitor and CYP2J2

Xiangge Tian [a#], Meirong Zhou [a #], Jing Ning [c #], Xiaopeng Deng[a], Lei Feng [c], Huilian Huang [a], Dahong Yao [b*] and Xiaochi Ma [a, *]

[a] Laboratory of Modern Preparation of TCM, Ministry of Education, Jiangxi University of Traditional Chinese Medicine, Nanchang 330004, China;

[b] School of Pharmaceutical Sciences, Shenzhen Technology University, Shenzhen, 518118, China.

[c] Dalian Key Laboratory of Metabolic Target Characterization and Traditional Chinese Medicine Intervention, Dalian Medical University, Dalian, 116044, China ;

*Corresponding authors: Xiaochi Ma (maxc1978@163.com) and Dahong Yao (yaohong@szu.edu.cn)

[#] The authors contributed equally.

1. ^{1}H and ^{13}C NMR of Compounds

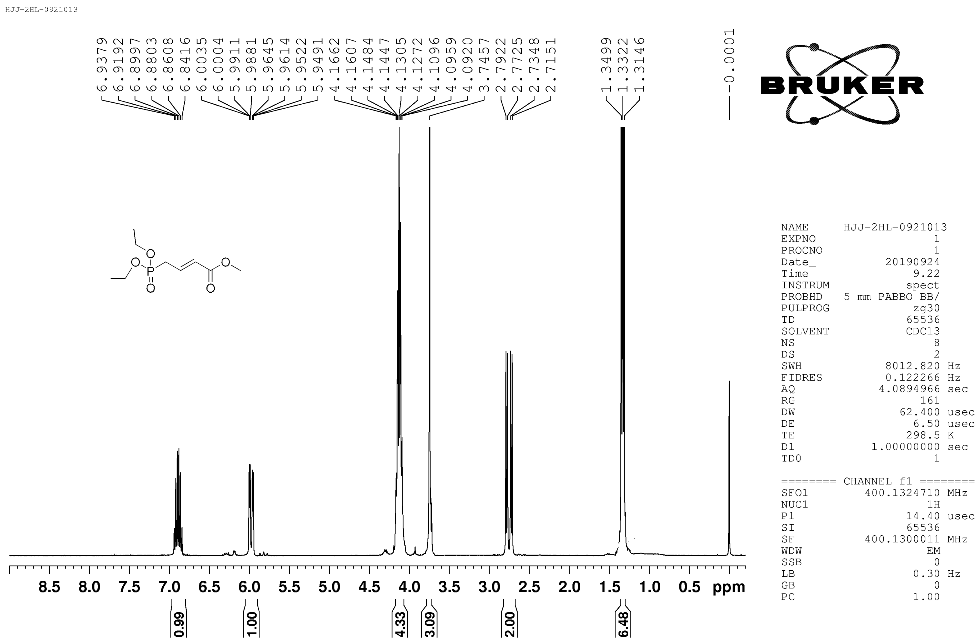

^{1}H-NMR spectrum of compound 3

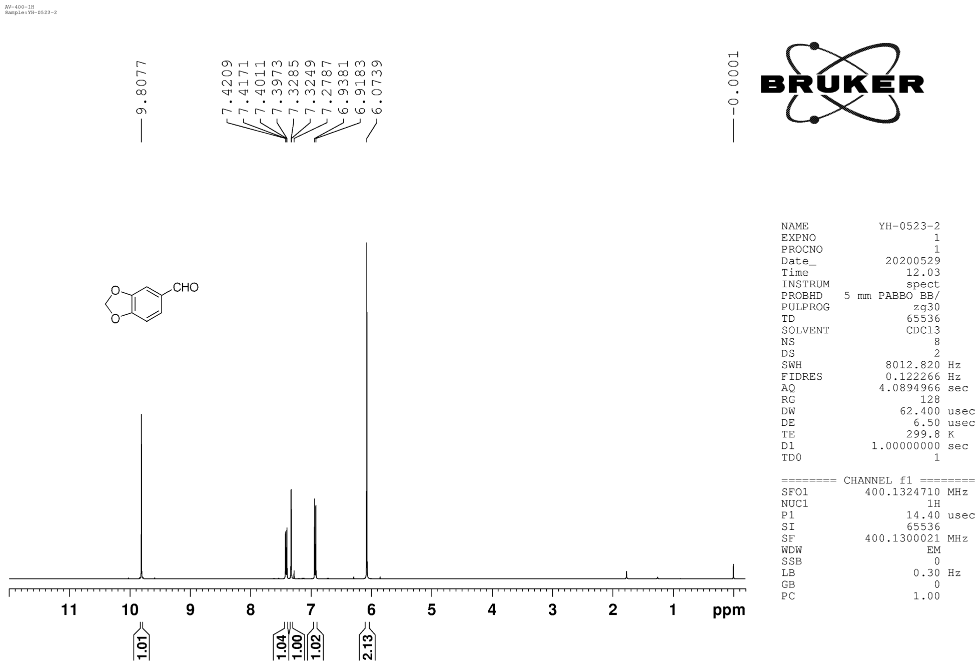

^{1}H-NMR spectrum of compound 6

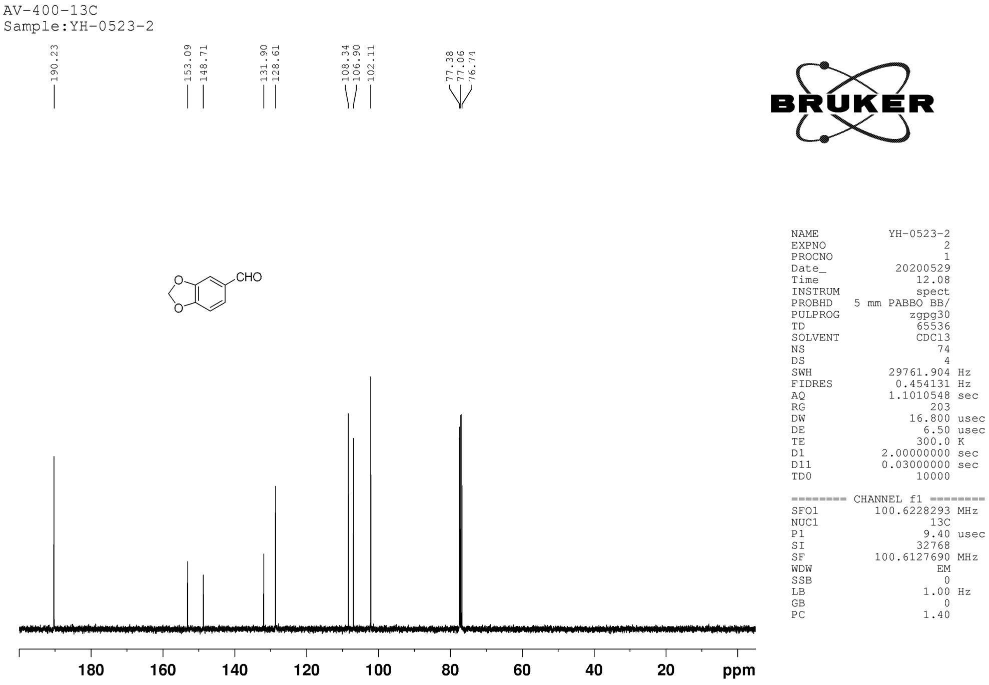

^{13}C-NMR spectrum of compound 6

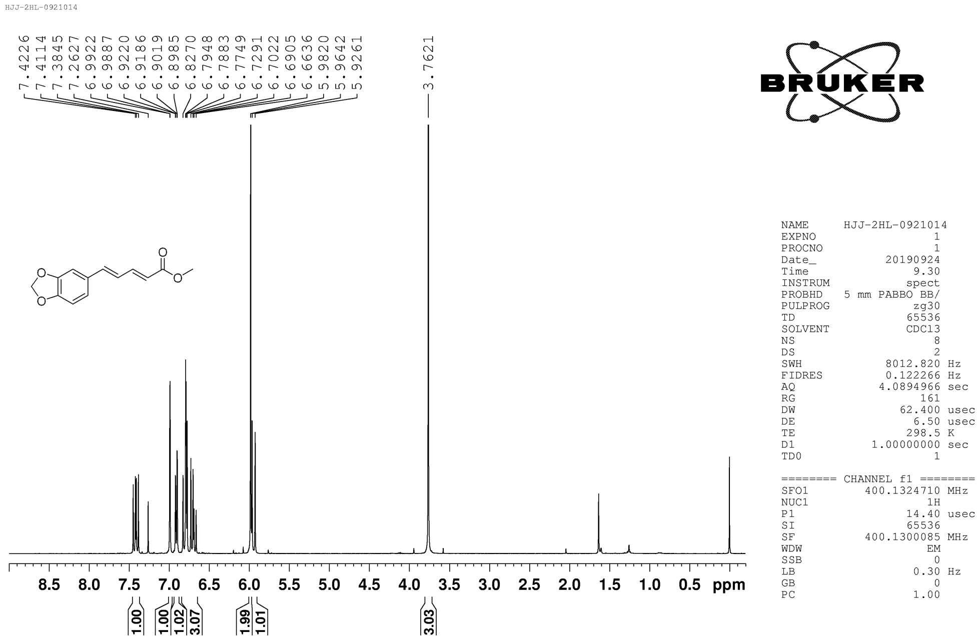

^{1}H-NMR spectrum of compound 7

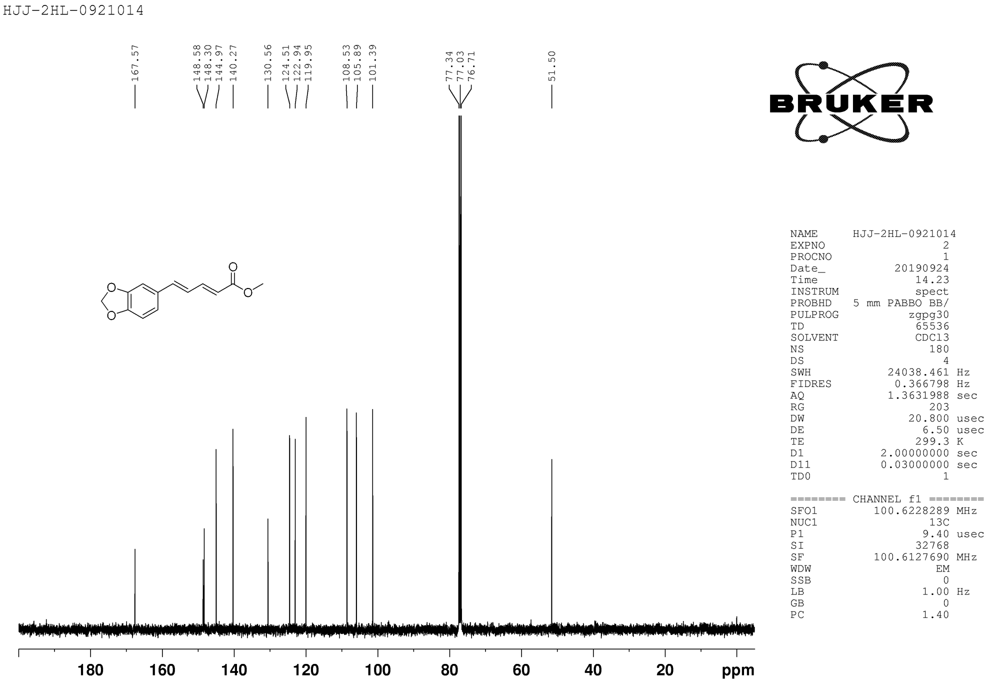

^{13}C-NMR spectrum of compound 7

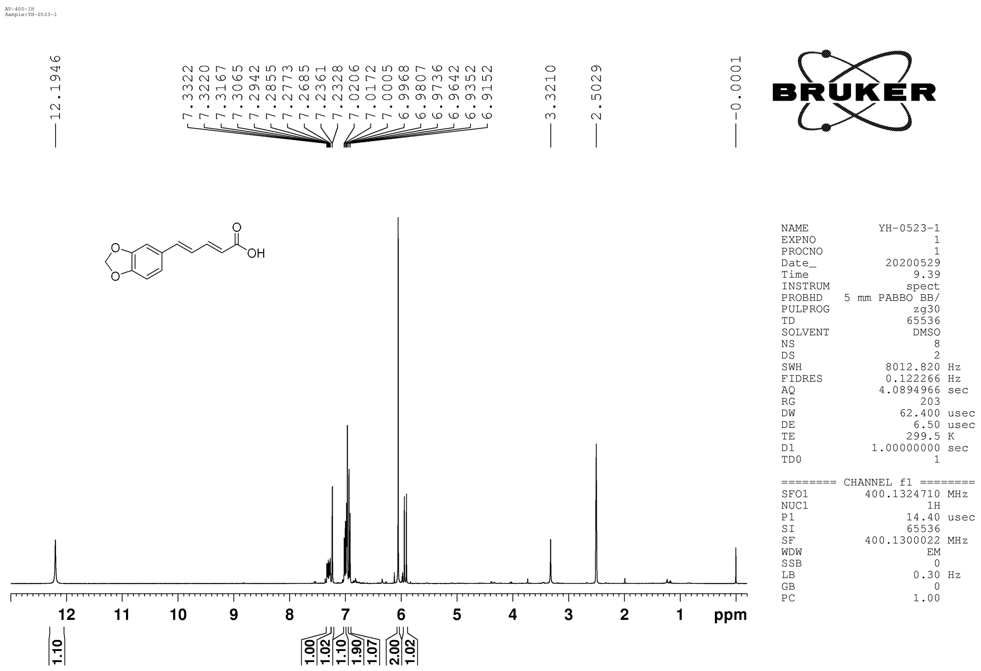

^{1}H-NMR spectrum of compound 8

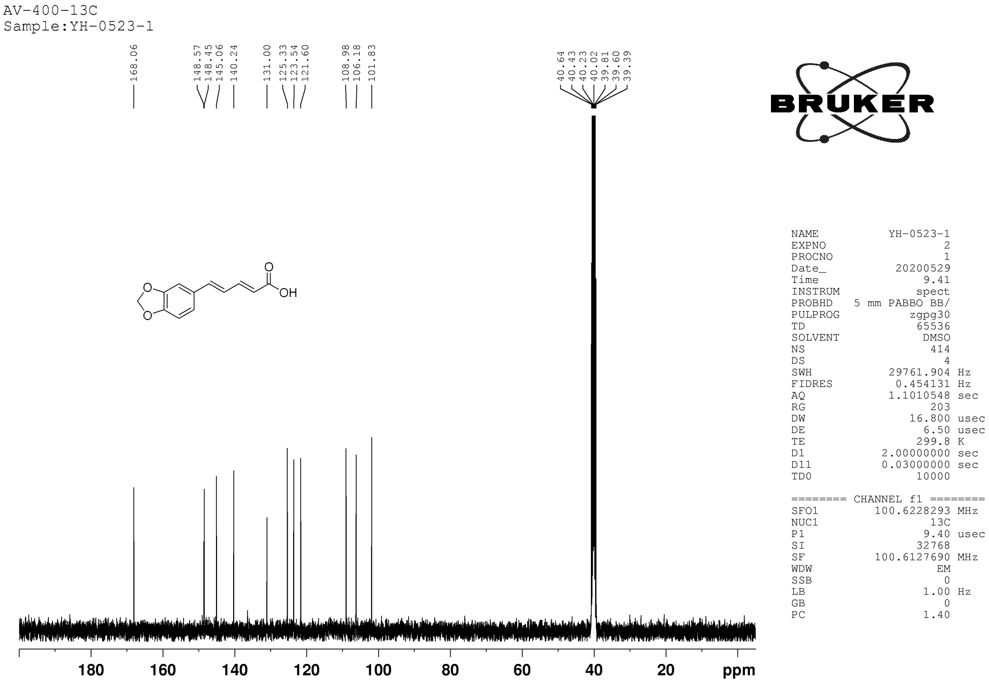

^{13}C-NMR spectrum of compound 8

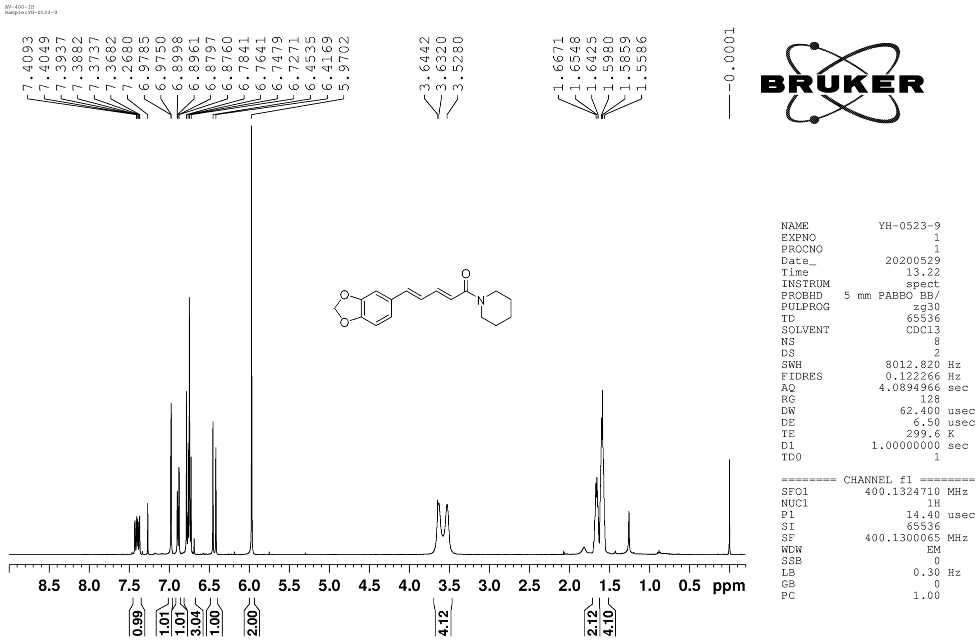

^{1}H-NMR spectrum of compound 9a

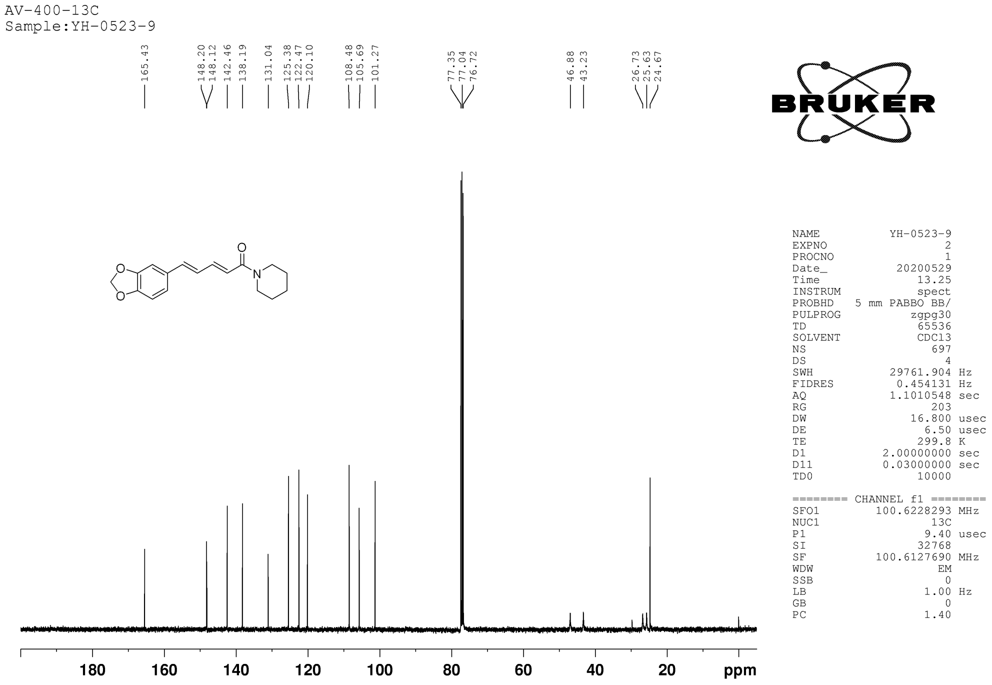

^{13}C-NMR spectrum of compound 9a

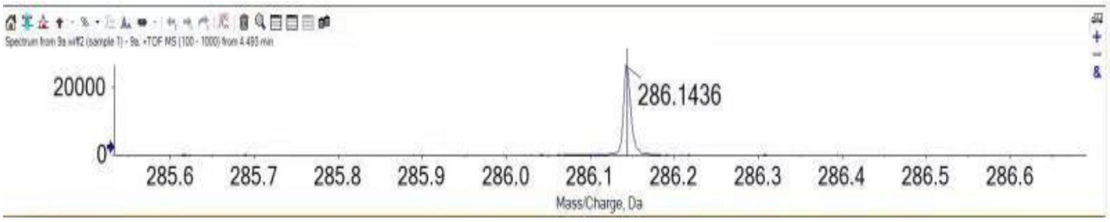

HRMS spectrum of compound 9a

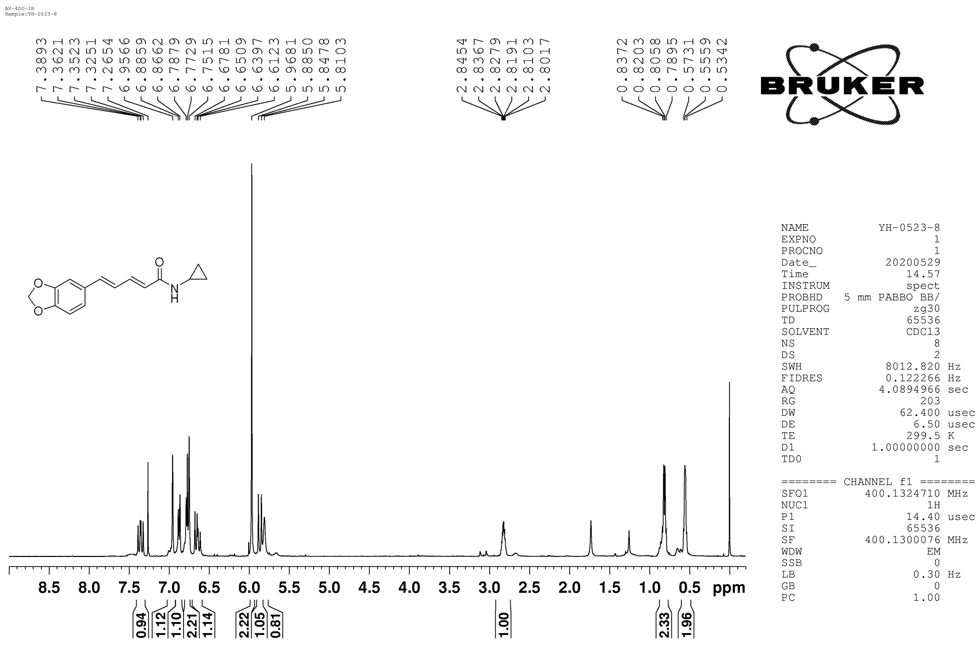

^{1}H-NMR spectrum of compound 9b

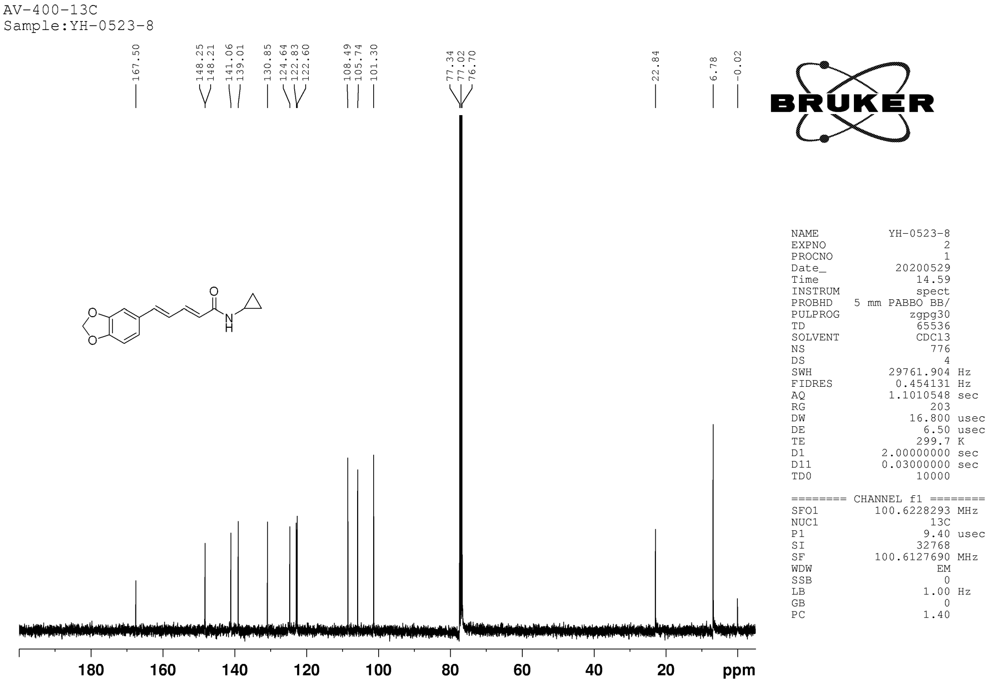

^{13}C-NMR spectrum of compound 9b

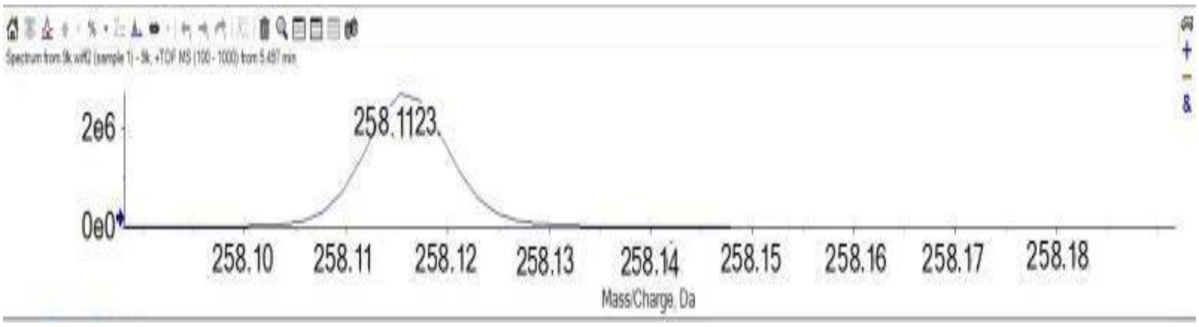

HRMS spectrum of compound 9b

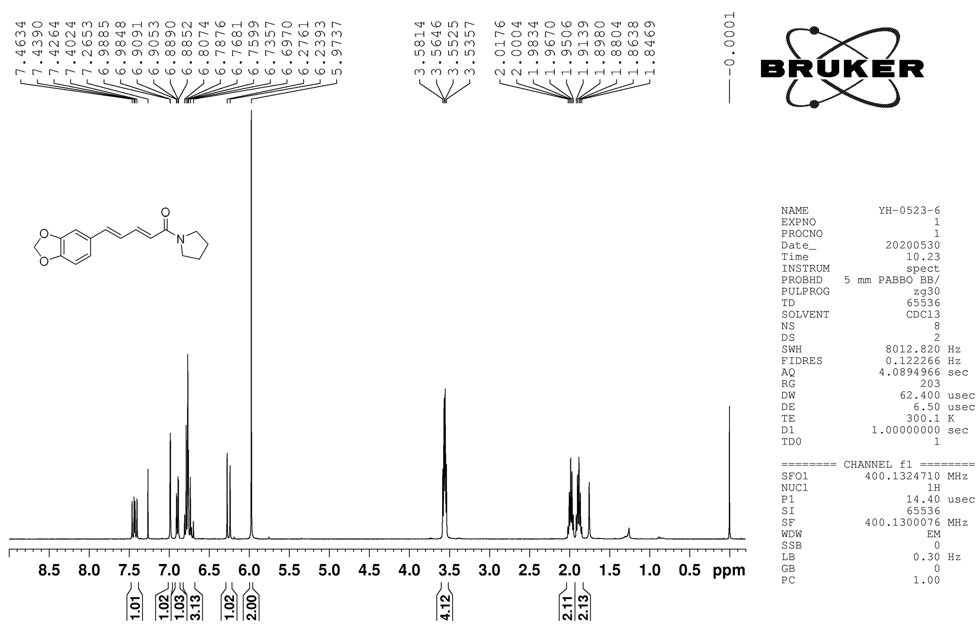

^{1}H-NMR spectrum of compound 9c

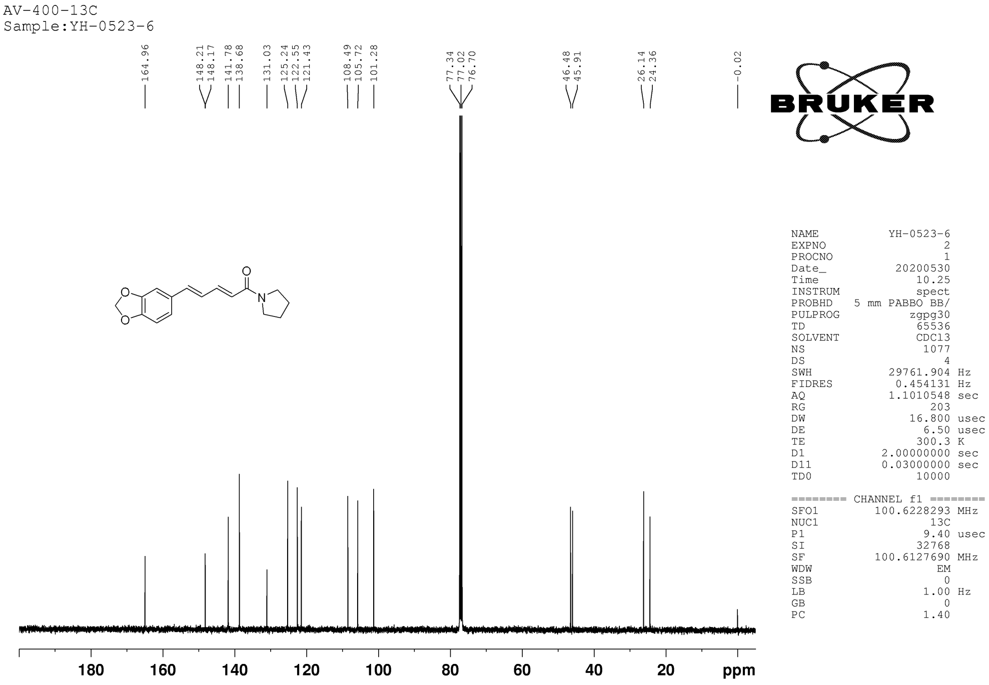

^{13}C-NMR spectrum of compound 9c

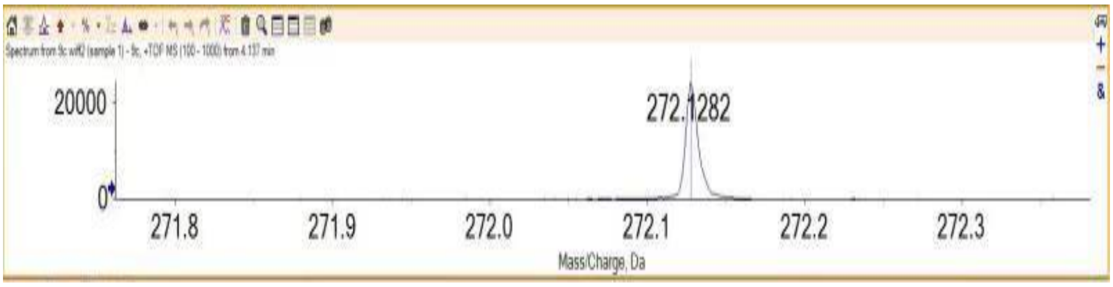

HRMS spectrum of compound 9c

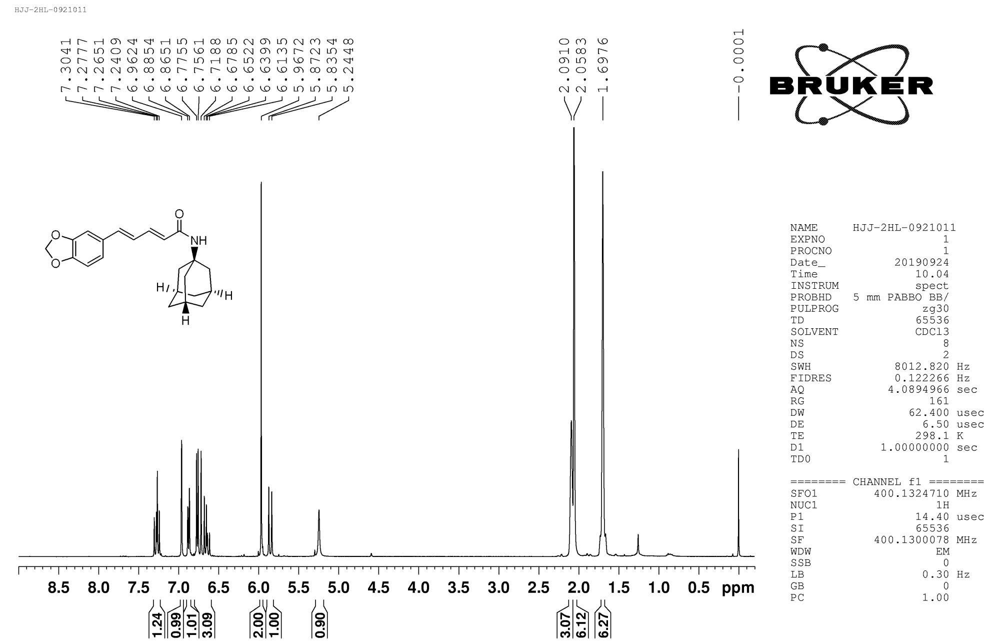

^{1}H-NMR spectrum of compound 9d

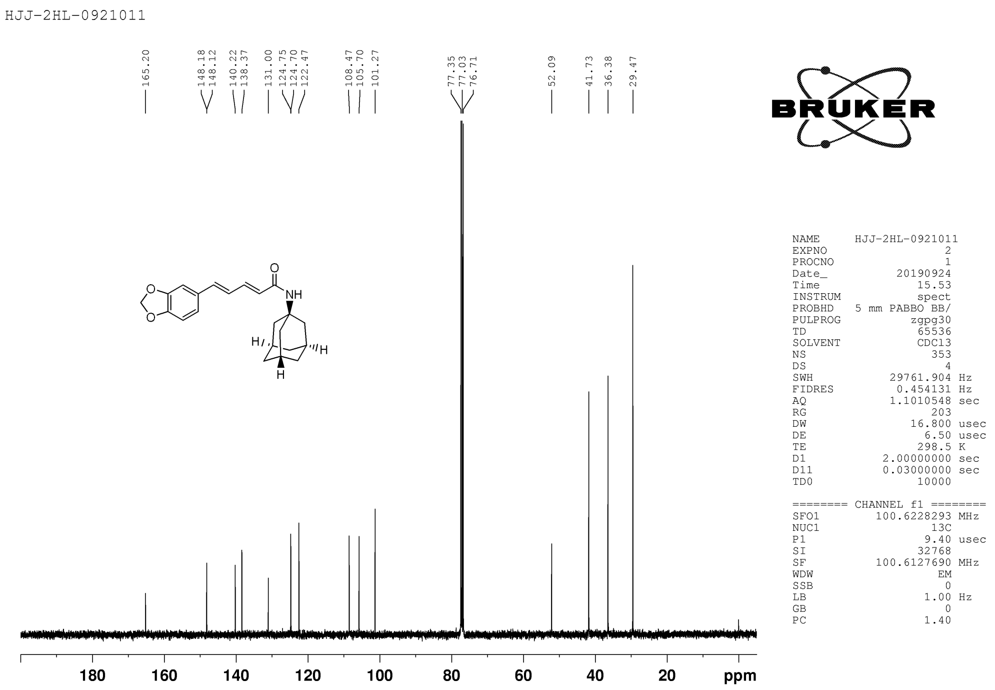

^{13}C-NMR spectrum of compound 9d

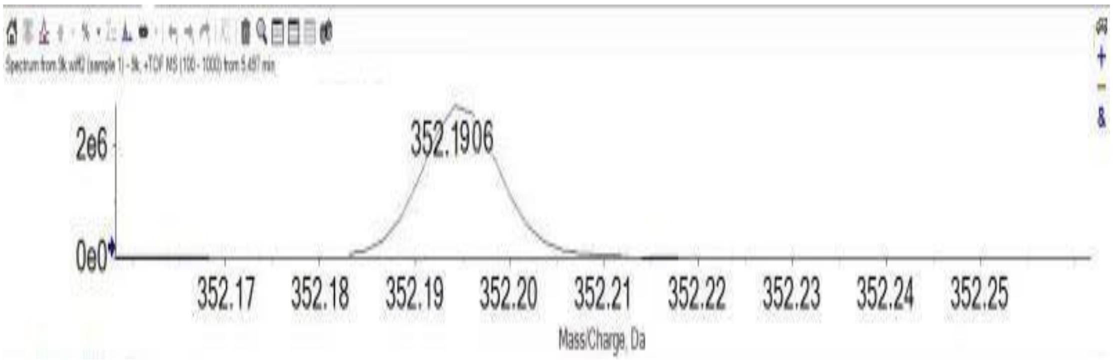

HRMS spectrum of compound 9d

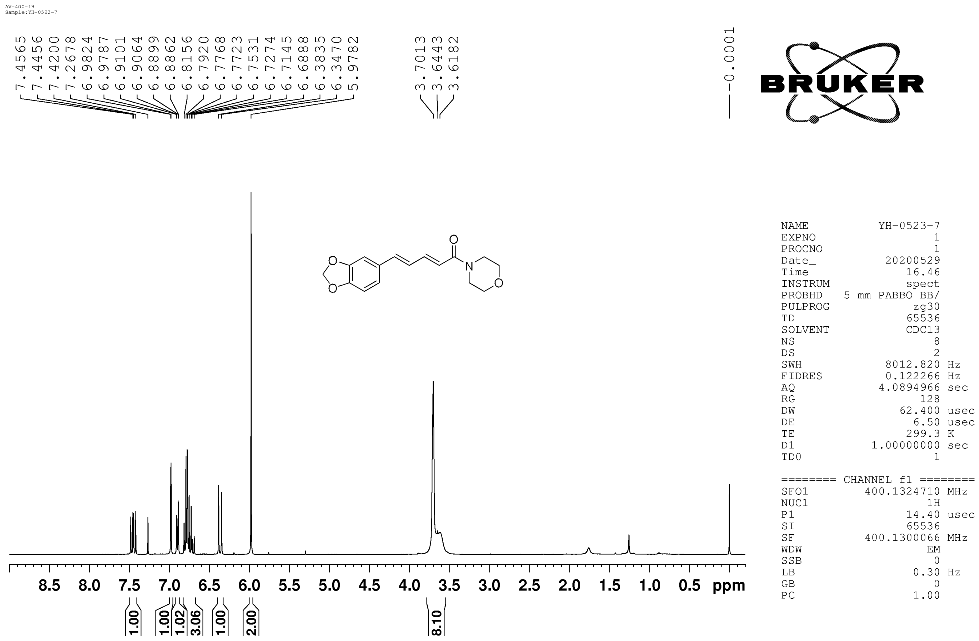

^{1}H-NMR spectrum of compound 9e

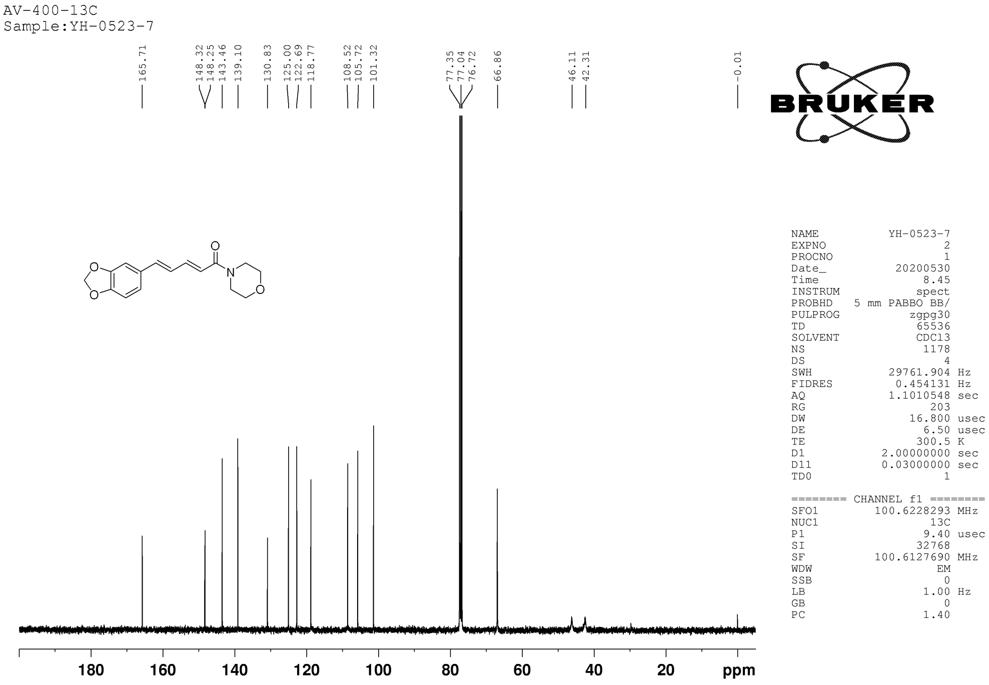

^{13}C-NMR spectrum of compound 9e

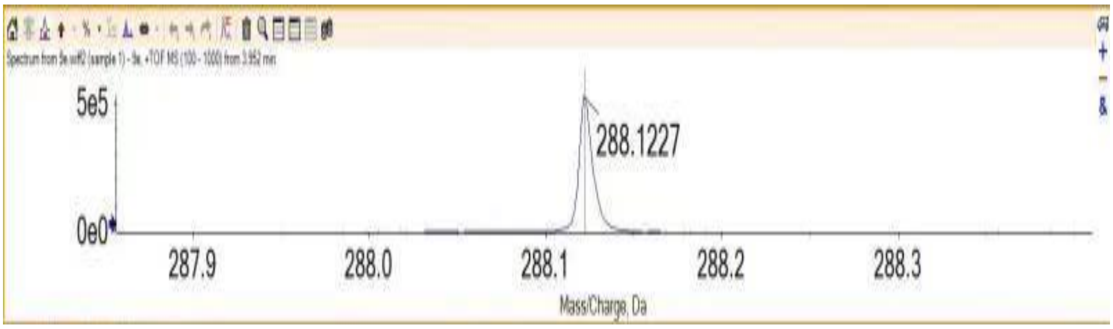

HRMS spectrum of compound 9e

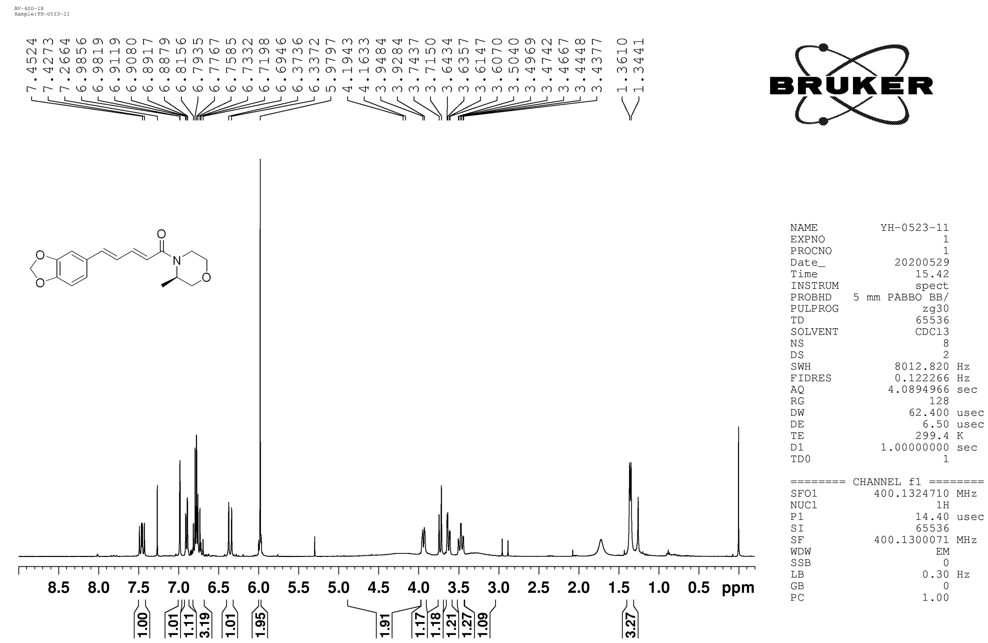

^{1}H-NMR spectrum of compound 9f

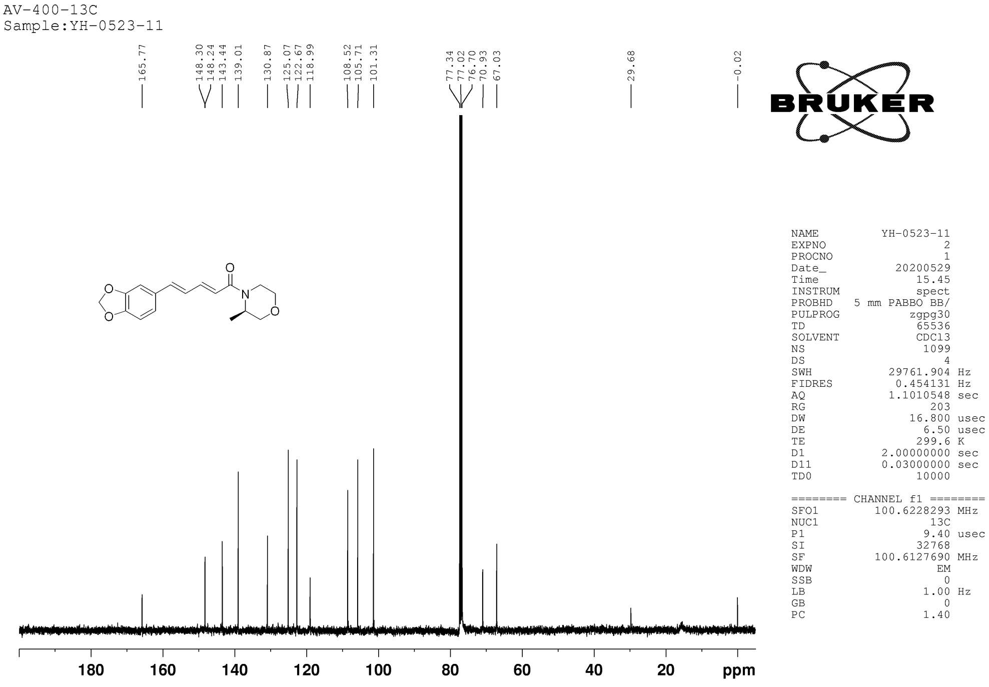

^{13}C-NMR spectrum of compound 9f

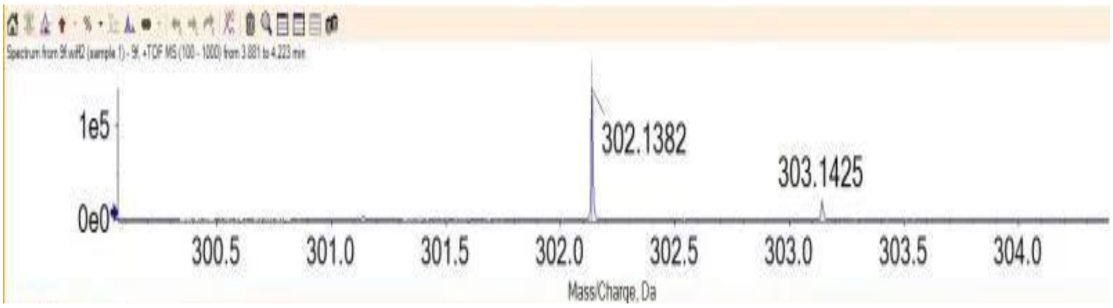

HRMS spectrum of compound 9f

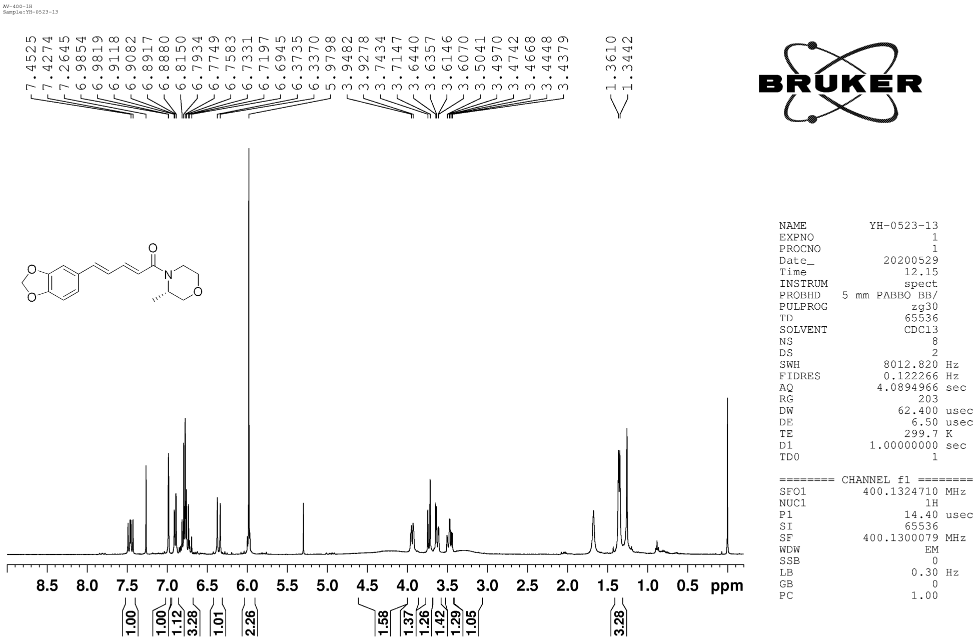

¹H-NMR spectrum of compound 9g

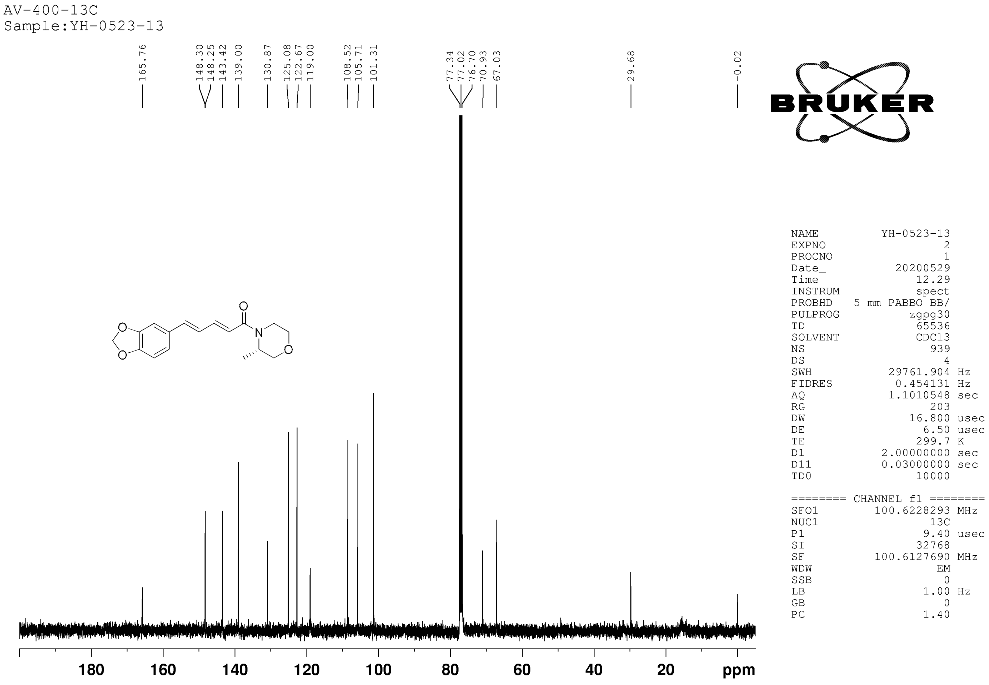

^{13}C-NMR spectrum of compound 9g

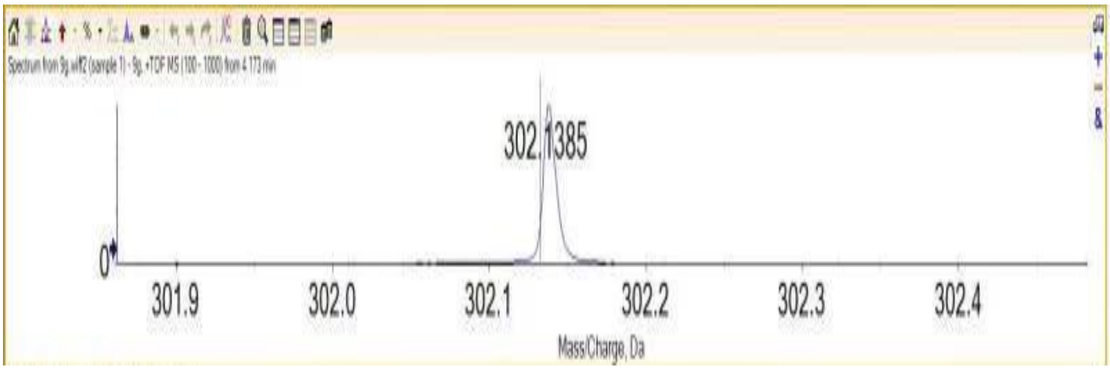

HRMS spectrum of compound 9g

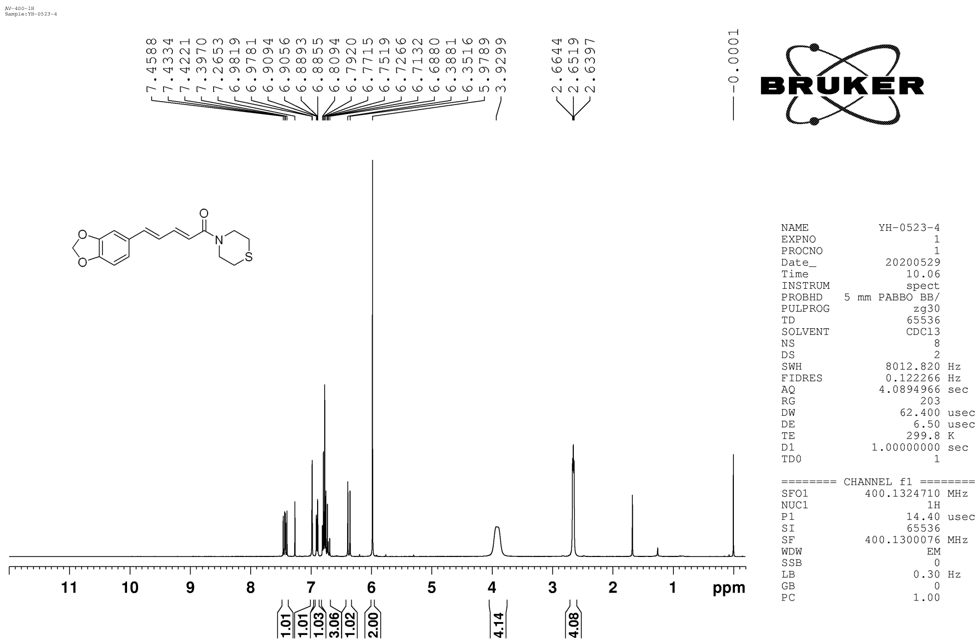

^{1}H-NMR spectrum of compound 9h

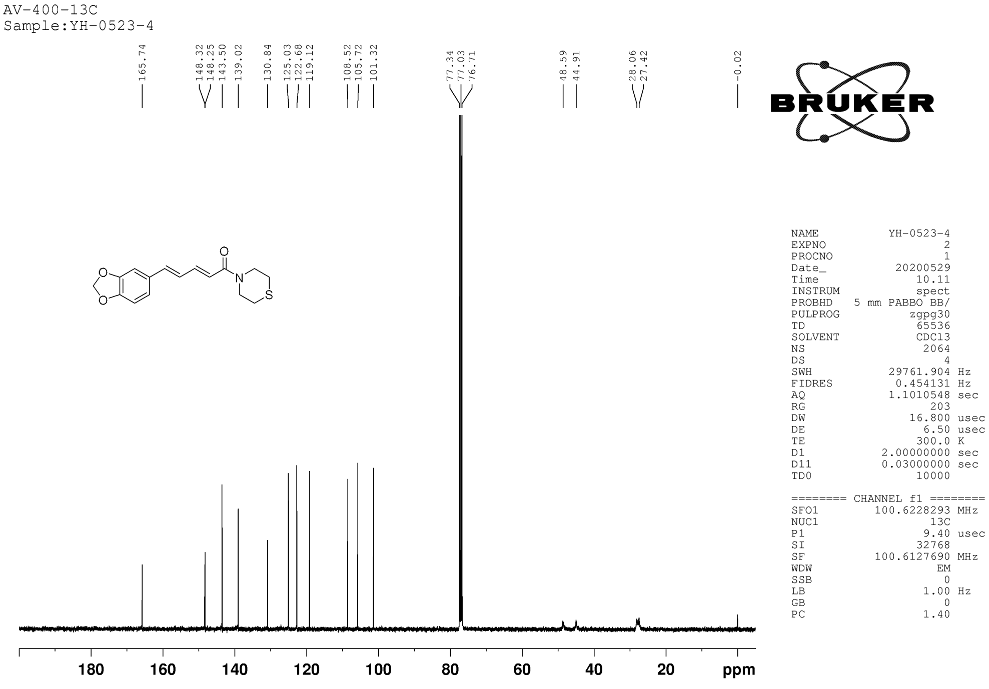

^{13}C-NMR spectrum of compound 9h

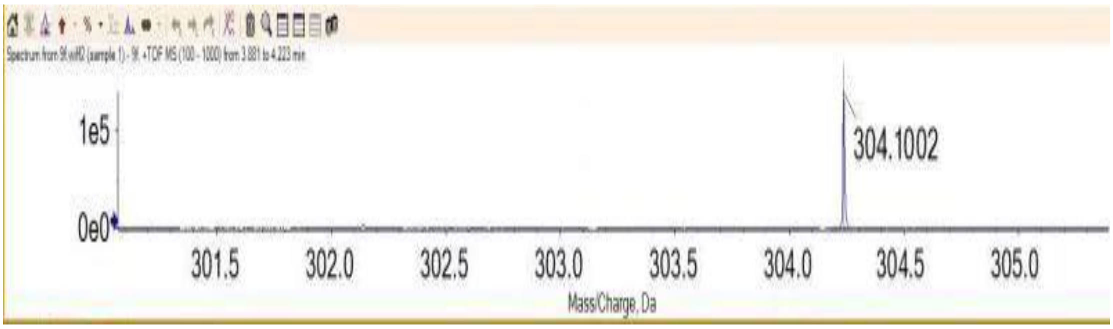

HRMS spectrum of compound 9h

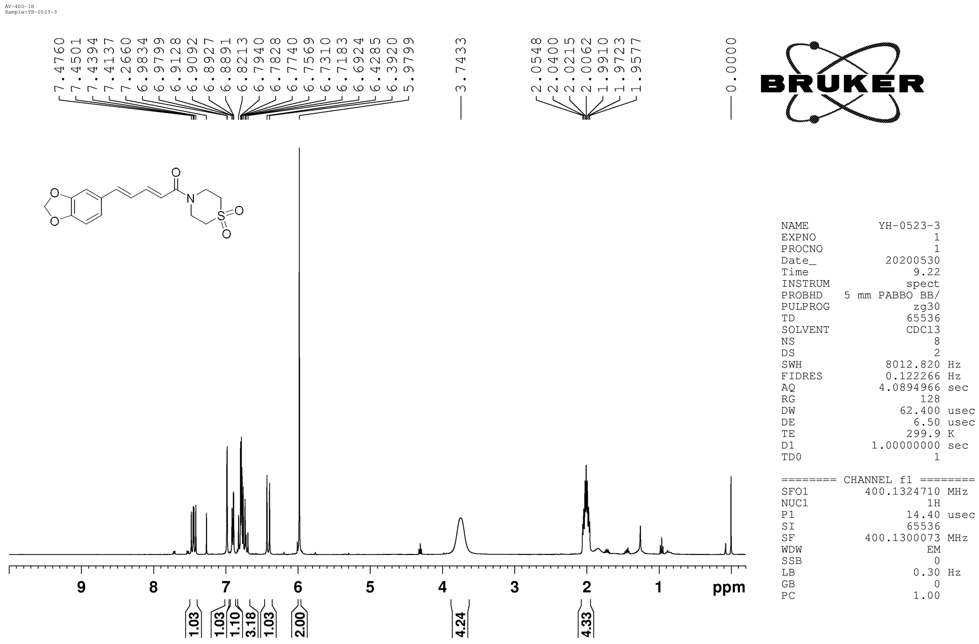

^{1}H-NMR spectrum of compound 9i

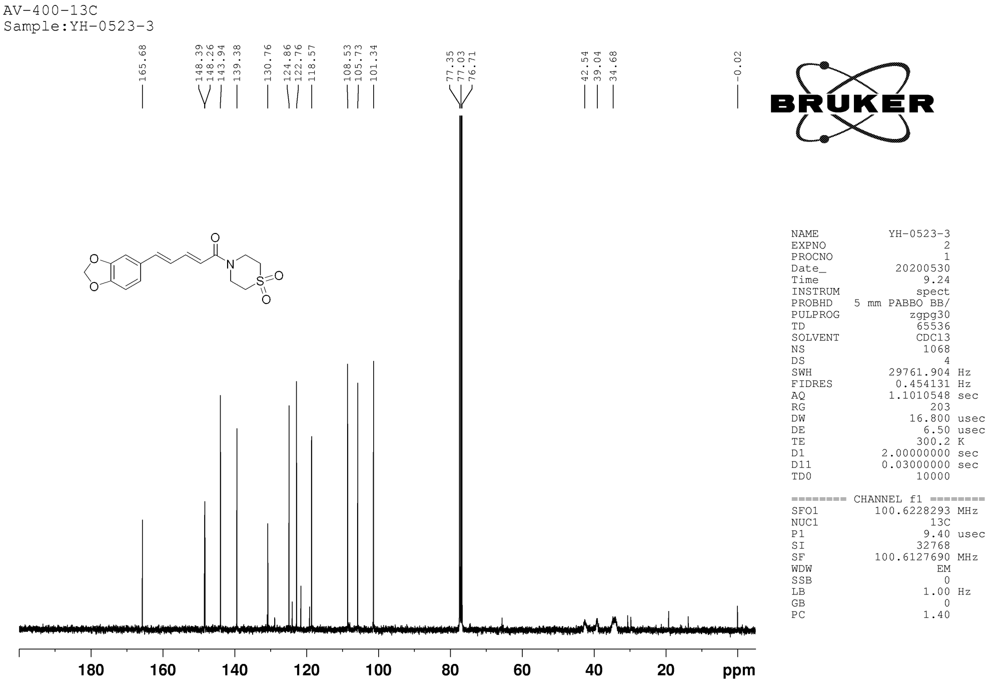

^{13}C-NMR spectrum of compound 9i

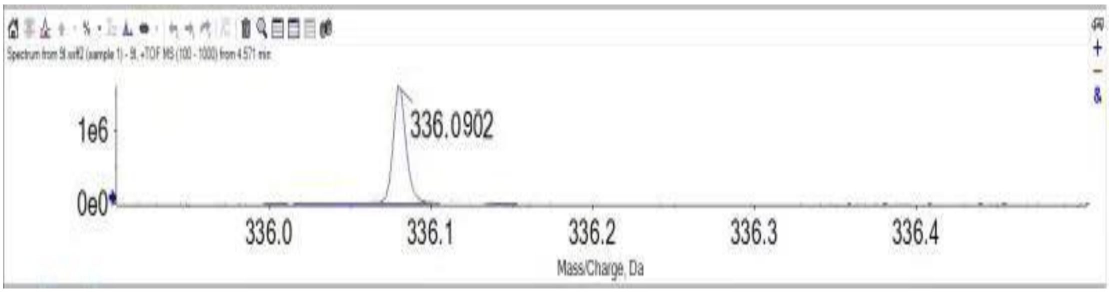

HRMS spectrum of compound 9i

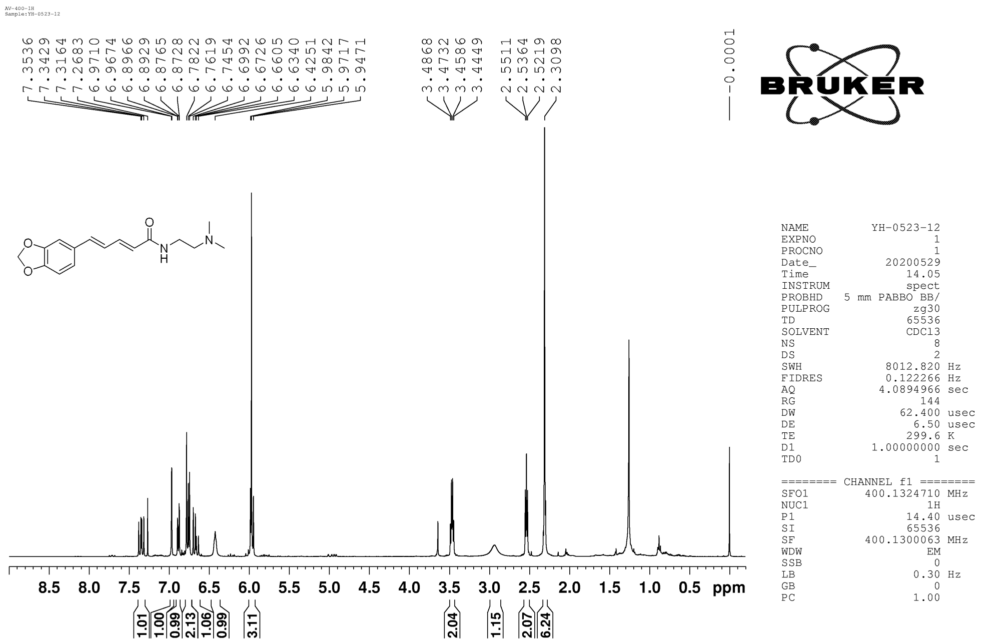

^{1}H-NMR spectrum of compound 9j

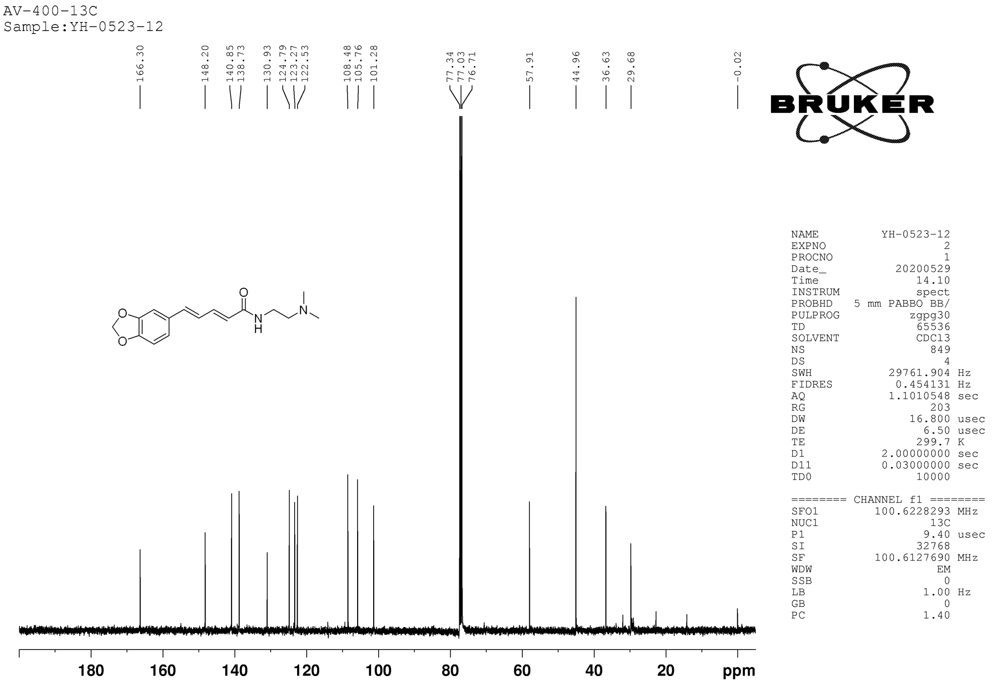

^{13}C-NMR spectrum of compound 9j

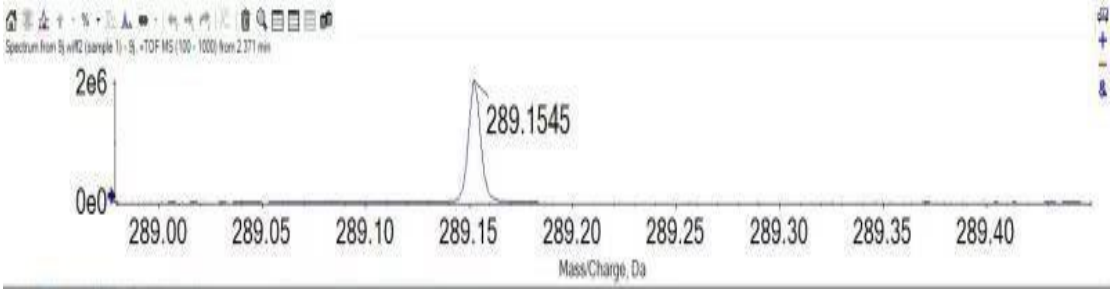

HRMS spectrum of compound 9j

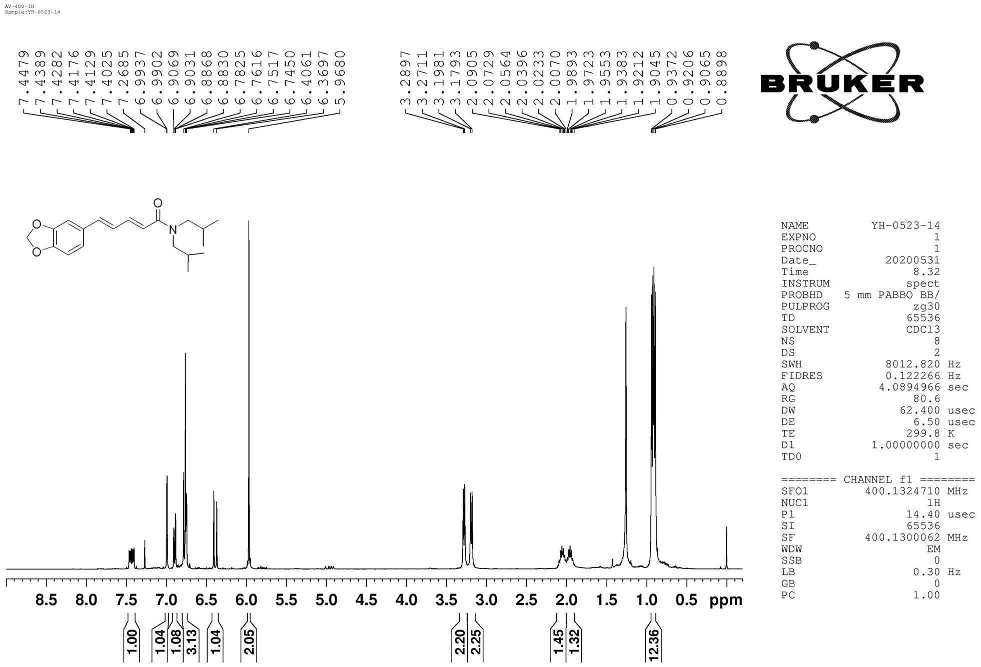

^{1}H-NMR spectrum of compound 9k

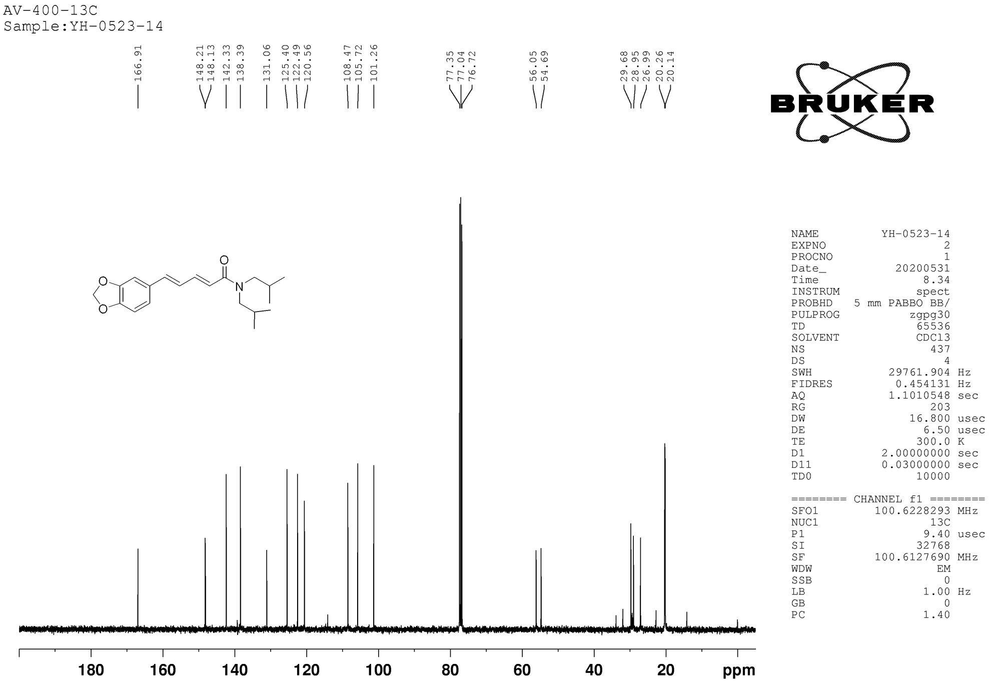

^{13}C-NMR spectrum of compound 9k

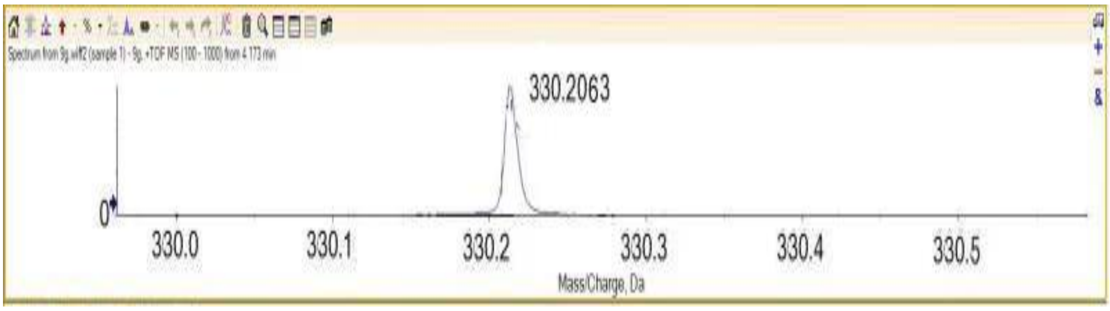

HRMS spectrum of compound 9k

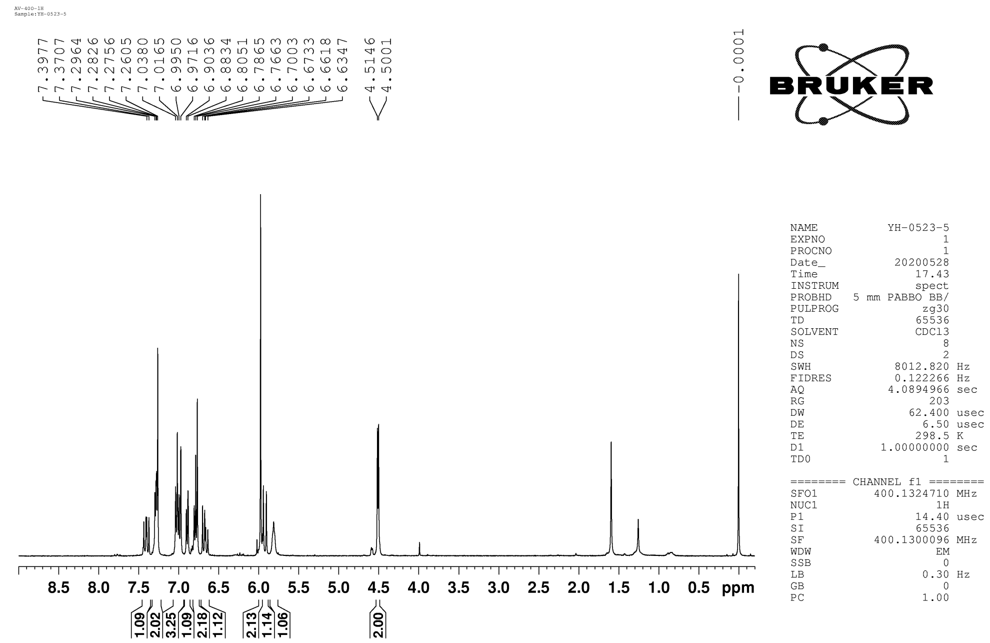

^{1}H-NMR spectrum of compound 9l

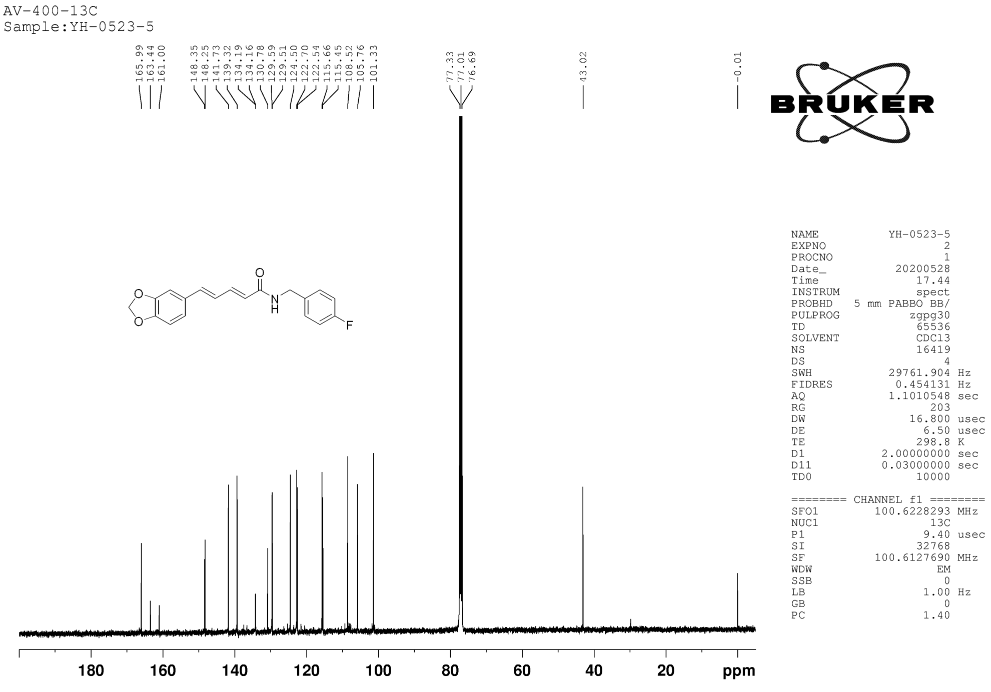

^{13}C-NMR spectrum of compound 9l

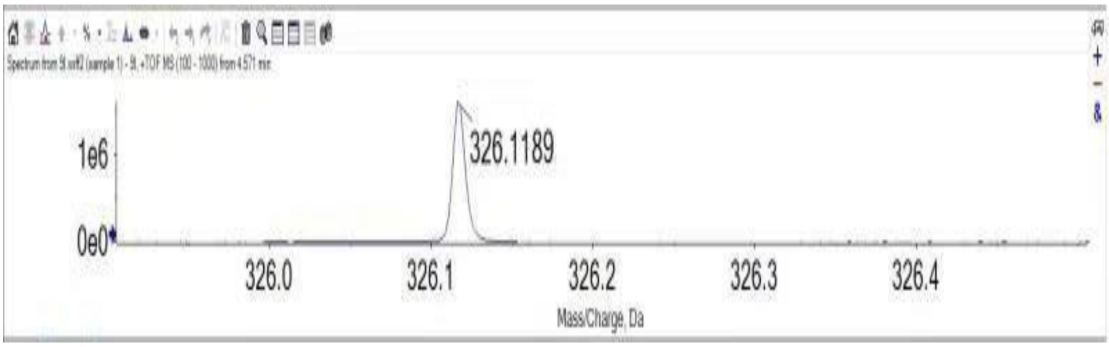

HRMS spectrum of compound 9l

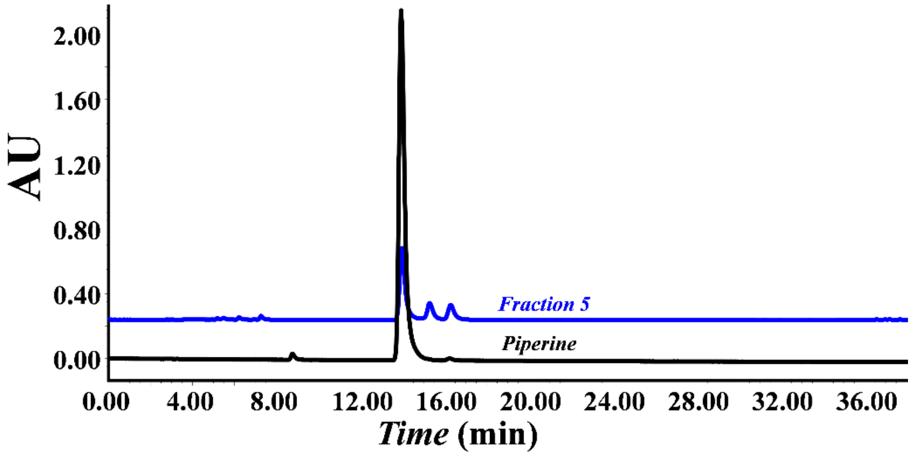

Fig. S1. The HPLC analysis of Fraction 5 from *Piper nigrum L.* of and Piperine.

Table S1 The name of 108 herbal medicines

No.	Name
1	*Cuscuta chinensis Lam.*
2	*Equisetum hyemale L.*
3	*Fructus Ligustri Lucidi*
4	*Artemisia argyi Levl.et Vant.*
5	*Portulaca oleracea L.*
6	*MeLia toosendanSieb.et Zucc.*
7	*Ephedra sinica Stapf*
8	*Diospyros kaki Thunb.*
9	*Agrimonia pilosa Ldb.*
10	*Euphorbiahumifusa Willd.*
11	*Astragalus complanatus R．Br.*
12	*Momordica cochinchinensis (Lour.) Spreng.*
13	*Quisqualis indica L.*
14	*Caulis Sinomenii*
15	*Tribulus terrester L.*
16	*Citrus medic L.*
17	*Dianthus superbus L．*
18	*Perillafrutescens*
19	*LonicerajaponicaThunb*
20	*Sparganium stoloni erum,Buch. -Ham.*
21	*Evodia rutaecarpa (Juss.) Benth.*
22	*Lysimachia christinae Hance*
23	*Platycladus orientalis*
24	*CaesalpiniasappanL.*
25	*Fructus viticis*
26	*Curcuma longa L.*
27	*Curcuma rcenyujin Y，H. Chenet C.Ling*
28	*Percarpium arecae catechu*
29	*Cynomorium songaricum Rupr.*
30	*Benincasa his pida (Thunb.) Cogn.*
31	*Vaccaria v. wolf*
32	*Aconitum gymnandrum Maxim.*
33	*Polygonum aviculare L.*
34	*Dolicho LablabL.*
35	*Achyranthes bidentata Blume.*
36	*Hordeurn vulgare L.*
37	*Cicadae Periostracum*
38	*Asarum sieboldii Miq.*
39	*Stemmacanthauniflora (L.) Dittrich*
40	*Radix Euphorbiae Ebracteolatae*
41	*Stephania tetrandra*

42	*Angelica sinensis*
43	*Angelica dahurica （Fisch. ex Hoffm.）Benth. et Hook. f. ex Franch. et Sav*
44	*Dryopteris crassirhizoma Nakai*
45	*Epimedium brevicornu Maxim.*
46	*Salvia miltiorrhiza Bge.*
47	*Pycnonotus sinensis*
48	*Scutellaria baicalensis Georgi*
49	*Citrus aurantium L.*
50	*Trichosanthes kirilowii Maxim.*
51	*Catsia tora Linn*
52	*Peucedanum praeruptorum Dunn*
53	*Magnolia officinalis Rehd. et Wils.*
54	*Euphorbia fischeriana Steud.*
55	*Platycodon grandiflorus (Jacq.) A. DC.*
56	*Paeonia suffruticosa Andr.*
57	*Cortex Fraxini*
58	*Aloe vera（Haw.） Berg*
59	*Psoralea corylifolia Linn.*
60	*Uncaria rhynchophylla（Miq.）Miq. ex Havil.*
61	*Reynoutria japonica Houtt.*
62	*Cinnamomum cassia Presl*
63	*Glycyrrhiza uralensis Fisch.*
64	*Atractylodes lancea(Thunb.)DC.*
65	*Sophora japonica Linn*
66	*Poria cocos(Schw.)Wolf*
67	*Fallopia multiflora (Thunb.) Harald*
68	*Houttuynia cordata Thunb.*
69	*Forsythia suspensa*
70	*Sophora flavescens Ait.*
71	*Boswellia carterii Birdw.*
72	*Piper Nigrum L.*
73	*Hibiscus syriacus L.*
74	*Realgar*
75	*Rehmannia glutinosa (Gaetn.) Libosch. ex Fisch. et Mey.*
76	*IsatisindigoticaFort.*
77	*Acorus tatarinowii*
78	*Eugenia caryophμllata*Thunb.
79	*Lithospermum erythrorhizon Sieb. et Zucc.*
80	*Andrographis paniculata*
81	*Cynanchum otophyllum Schneid.*
82	*Acanthopanax senticosus (Rupr. Maxim.) Harms*
83	*Dendranthema indicum*
84	*Smilax china L.*
85	*Ginkgo biloba L.*

86	*Glycyrrhizae.*
87	*Flos Sophorae Immaturus*
88	*Mentha spicata Linn.*
89	*Leonurus japonicus Houtt*
90	*Dendranthema morifolium (Ramat.) Tzvelev*
91	*Alisma plantago-aquatica Linn.*
92	*Dendrobium nobile Lindl*
93	*Rubia cordifolia L.*
94	*Dioscorea oppositifolia L.*
95	*Artemisia capillaris Thunb.*
96	*Saposhnikovia divaricata (Trucz.) Schischk.*
97	*FoliumSennae*
98	*Arctium lappa L.*
99	*Gardenia jasminoides Ellis*
100	*Radix Bupleuri*
101	*Notopterygium incisum Ting ex H.T.Chang*
102	*Belamcanda chinensis （L.）Redouté*
103	*Angelica pubescens Maxim.f. biserrata Shan et Yuan*
104	*Adenophor acapillaris Hemsl.*
105	*Citrus reticulata Blanco*
106	*Radix Aucklandiae*
107	*Rheum palmatum L.*
108	*Lonicera japonica Thunb.*
